# Osteoclasts recycle via osteomorphs during RANKL-stimulated bone resorption

**DOI:** 10.1016/j.cell.2021.02.002

**Published:** 2021-03-04

**Authors:** Michelle M. McDonald, Weng Hua Khoo, Pei Ying Ng, Ya Xiao, Jad Zamerli, Peter Thatcher, Wunna Kyaw, Karrnan Pathmanandavel, Abigail K. Grootveld, Imogen Moran, Danyal Butt, Akira Nguyen, Sean Warren, Maté Biro, Natalie C. Butterfield, Siobhan E. Guilfoyle, Davide Komla-Ebri, Michael R.G. Dack, Hannah F. Dewhurst, John G. Logan, Yongxiao Li, Sindhu T. Mohanty, Niall Byrne, Rachael L. Terry, Marija K. Simic, Ryan Chai, Julian M.W. Quinn, Scott E. Youlten, Jessica A. Pettitt, David Abi-Hanna, Rohit Jain, Wolfgang Weninger, Mischa Lundberg, Shuting Sun, Frank H. Ebetino, Paul Timpson, Woei Ming Lee, Paul A. Baldock, Michael J. Rogers, Robert Brink, Graham R. Williams, J.H. Duncan Bassett, John P. Kemp, Nathan J. Pavlos, Peter I. Croucher, Tri Giang Phan

**Affiliations:** 1 Healthy Ageing Theme, Garvan Institute of Medical Research, Sydney, NSW, Australia; 2 St. Vincent’s Clinical School, Faculty of Medicine, UNSW Sydney, NSW, Australia; 3 Bone Biology & Disease Laboratory, School of Biomedical Sciences, University of Western Australia, Nedlands, WA, Australia; 4 Immunology Theme, Garvan Institute of Medical Research, Sydney, NSW, Australia; 5 Cancer, Garvan Institute of Medical Research, Sydney, NSW, Australia; 6 EMBL Australia, Single Molecule Science Node, School of Medical Sciences, University of New South Wales, Sydney, NSW, Australia; 7 Molecular Endocrinology Laboratory, Department of Metabolism, Digestion & Reproduction, Imperial College London, London, UK; 8 John Curtin School of Medical Research, The Australian National University, Canberra, ACT, Australia; 9 Immune Imaging Program, Centenary Institute, Sydney, NSW, Australia; 10 Sydney Medical School, University of Sydney, Sydney, NSW, Australia; 11 Department of Dermatology, Medical University of Vienna, Vienna, Austria; 12 The University of Queensland Diamantina Institute, University of Queensland, Woolloongabba, QLD, Australia; 13 Transformational Bioinformatics, Commonwealth Scientific and Industrial Research Organisation, Sydney, NSW, Australia; 14 Biovinc, Pasadena, CA, USA; 15 Medical Research Council Integrative Epidemiology Unit, University of Bristol, Bristol, UK

**Keywords:** osteomorph, osteoclast, cell fission, cellular recycling, macrophage, RANKL, osteoprotegerin, denosumab, osteoporosis, skeletal dysplasia

## Abstract

Osteoclasts are large multinucleated bone-resorbing cells formed by the fusion of monocyte/macrophage-derived precursors that are thought to undergo apoptosis once resorption is complete. Here, by intravital imaging, we reveal that RANKL-stimulated osteoclasts have an alternative cell fate in which they fission into daughter cells called osteomorphs. Inhibiting RANKL blocked this cellular recycling and resulted in osteomorph accumulation. Single-cell RNA sequencing showed that osteomorphs are transcriptionally distinct from osteoclasts and macrophages and express a number of non-canonical osteoclast genes that are associated with structural and functional bone phenotypes when deleted in mice. Furthermore, genetic variation in human orthologs of osteomorph genes causes monogenic skeletal disorders and associates with bone mineral density, a polygenetic skeletal trait. Thus, osteoclasts recycle via osteomorphs, a cell type involved in the regulation of bone resorption that may be targeted for the treatment of skeletal diseases.

## Highlights

• Osteoclasts fission into daughter cells called osteomorphs
• Osteomorphs fuse and recycle back into osteoclasts
• Osteomorph upregulated genes control bone structure and function in mice
• Osteomorph upregulated genes are implicated in rare and common bone diseases in humans

## Graphical abstract

**Figure:**
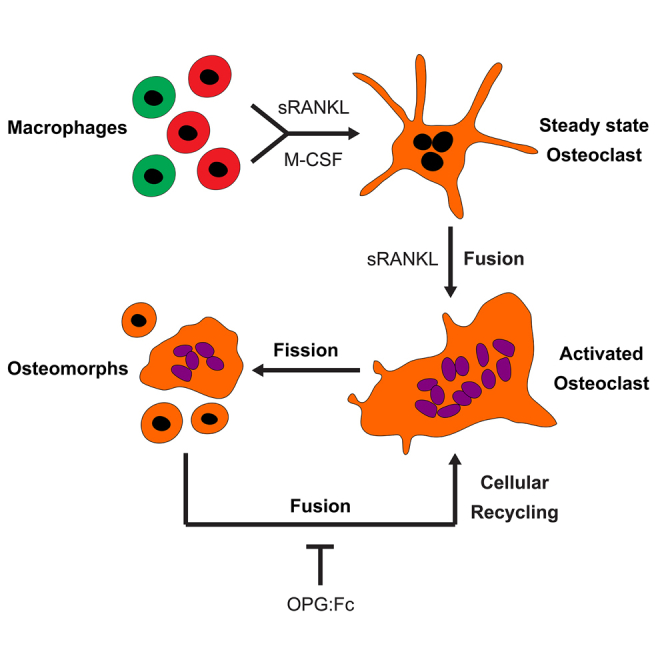

## Teaser

Tracking osteoclasts during cycles of fission and fusion reveals a transcriptionally distinct “osteomorph” population that are fusion competent, motile, and capable of forming osteoclasts that resorb bone.

## Introduction

The skeleton provides the scaffold to support body weight, enable body movement, protect vital organs, and control mineral homeostasis while also providing the location for hematopoiesis. Accordingly, it is a dynamic organ that is continuously remodeled throughout life in response to diverse environmental stimuli. At the microscopic level, remodeling is achieved by the coordinated action of osteoclasts that resorb old bone and osteoblasts that form new bone, activities that are coupled in both time and space (Hattner et al., 1965). Osteoclasts are specialized cells formed by the fusion of committed monocyte/macrophage hematopoietic lineage precursor cells (Boyle et al., 2003; Ikeda and Takeshita, 2016). The importance of osteoclasts in bone homeostasis and human health is evidenced by the diseases in which osteoclast formation and function is dysregulated, such as Paget’s disease, osteoporosis, rheumatoid arthritis, and metastatic cancer (Novack and Teitelbaum, 2008). Notwithstanding the pivotal role of osteoclasts in health and disease, the biology of this cell type, particularly their life cycle *in vivo*, remains elusive.

Bone is opaque and has a high refractive index, making it difficult to image bone cells in live animals. Tracking osteoclast cell formation and fate in their native environment *in vivo* has proved demanding. As a result, advances in understanding osteoclast biology have largely been gained through study *in vitro*. This has been driven by the discovery that osteoclast-like cells could be generated by culture of bone marrow cells in the presence of vitamin D and parathyroid hormone (Takahashi et al., 1988). Subsequently, *in vitro* studies have contributed much to our understanding of the cellular origin of osteoclasts and the factors required for osteoclast formation and function, such as macrophage colony-stimulating factor (M-CSF), also known as colony stimulating factor-1 (CSF-1) (Udagawa et al., 1990), and what is now regarded as the critical osteoclast factor, the receptor activator of nuclear factor kappa B ligand (RANKL) (Boyce and Xing, 2007; Yasuda et al., 1998). Additionally, it has been shown that osteoclasts may undergo apoptosis as a pathological response to bisphosphonates (Hughes et al., 1995), estrogen (Nakamura et al., 2007), and mechanical force (Kobayashi et al., 2000). Collectively, this has led to the dogma that osteoclasts are terminally differentiated cells that undergo apoptosis after a short lifespan of ∼2 weeks (Manolagas, 2000). However, this has been challenged by a recent parabiosis study that showed osteoclasts are longer lived with a lifespan of around 6 months (Jacome-Galarza et al., 2019).

Two-photon microscopy, by imaging fluorescence excitation at near-infrared wavelengths that penetrate deep inside living tissues, has been used to image osteoclast precursors (Ishii et al., 2009; Ishii et al., 2010), osteoclastic bone resorption (Kikuta et al., 2013; Maeda et al., 2016), and osteoclast-osteoblast interactions *in vivo* (Furuya et al., 2018). These pioneering experiments were performed by visualizing cells expressing fluorescent reporters in the calvarium, a non-weight bearing flat bone formed by intramembranous ossification, that can be imaged via gaps in the cranial sutures in the bregma. Despite these studies, there have been no studies to date that have imaged osteoclast formation and fate *in vivo* in weight-bearing long bones formed by endochondral ossification.

To address this, we developed an alternative, minimally invasive system for longitudinal real-time imaging of cellular dynamics in the tibia of mice (Lawson et al., 2015). The tibia, unlike the calvaria, is a weight-bearing bone formed by endochondral ossification. It is a site of hematopoiesis and active bone turnover and models many of the bones that are commonly associated with skeletal disease. We leveraged knowledge that osteoclasts are formed by the fusion of cells of the monocyte/macrophage lineage to generate mixed bone marrow irradiation chimeras in which osteoclasts are marked by the simultaneous expression of two independent fluorescent reporters. This high-fidelity system for imaging osteoclast dynamics allowed us to visualize the behavior of these cells in the steady state upon activation with soluble RANKL (sRANKL) and upon inhibition with osteoprotegerin-Fc fusion protein (OPG:Fc), a reversible inhibitor of RANK signaling. These studies reveal an unexpected cell fate in which osteoclasts recycle by fissioning into smaller more motile cells, which we have termed osteomorphs, and then fusing to form osteoclasts in a different location. Osteomorphs were shown by single-cell RNA sequencing (scRNA-seq) to be a distinct cell type that differs from both osteoclasts and macrophages. Deletion of osteomorph upregulated genes in mice resulted in abnormal structural and functional bone phenotypes. Furthermore, human orthologs of osteomorph upregulated genes were associated with skeletal dysplasias and bone mineral density (BMD) in the UK Biobank study. Furthermore, we show that OPG:Fc treatment results in accumulation of osteomorphs that are able to reassemble rapidly into large activated osteoclasts upon OPG:Fc withdrawal, resulting in bone loss. These findings mirror the accelerated bone loss and spontaneous fractures recently described following discontinuation of treatment with denosumab, a monoclonal antibody that blocks RANK/RANKL signaling. Osteoclast recycling therefore not only provides a paradigm for understanding the behavior and fate of these cells in their physiological niche *in vivo* but also a framework for understanding skeletal diseases associated with dysregulated bone resorption and the effects of drugs used to treat them.

## Results

### Morphology and dynamics of osteoclasts in the steady state *in vivo*

**Figure 1:**
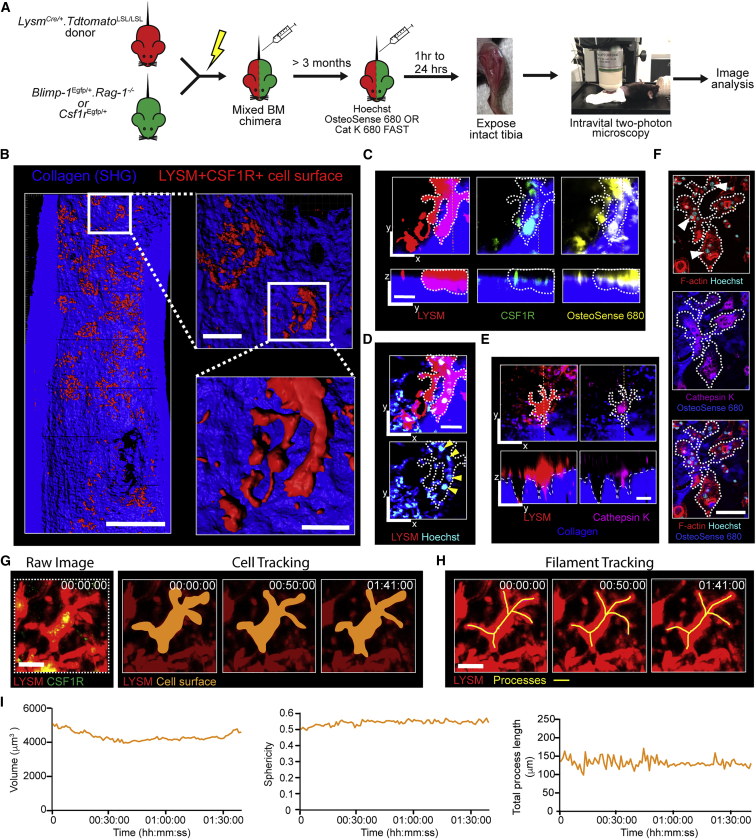
Intravital imaging of steady-state osteoclast dynamics (A) Schematic showing the method for tracking osteoclasts by intravital two-photon microscopy in mixed bone marrow chimeras. (B) 3D volume rendered image of the tibia showing the endosteal bone surface (blue, second harmonic generation, SHG) and the network of interconnecting osteoclasts (red pseudocolor). See also Video S1. Scale bars, 600 μm (tiled image, left) and 40 μm (inset image and bottom right). (C) Expression of LYSM (red), CSF1R (green), and uptake of OsteoSense 680 (yellow) by an osteoclast (dotted line) on the bone surface (blue). Scale bar, 50 μm. (D) Hoechst staining (cyan) showing multiple nuclei (yellow arrow heads) in the cell from (C). Scale bar, 50 μm. (E) Cathepsin K activity (magenta, right) in a large stellate osteoclast (red, left) on bone (blue). Scale bar, 50 μm. (F) Staining of *in vitro* generated osteoclast for Hoechst (cyan, white arrows), F-actin (red), cathepsin K activity (magenta), and OsteoSense 680 (blue). Scale bar, 25 μm. (G) Cell tracking using the surfaces feature in Imaris. Raw image (left panel) show LYSM^+^ (red) and CSF1R^+^ (green) cells. Time-lapse (right panels) show tracked double-labeled osteoclast. Scale bar, 25 μm. Timestamp hh:mm:ss. (H) Tracking morphology using the FilamentTracer plug-in in Imaris of osteoclast from (G). Cell processes shown by yellow lines. (I) Dynamic changes in cell volume (left), sphericity (middle), and total process length (right) of the cell tracked in (G and H). See also Video S2.

The expression of genes such as *Ctsk* (encoding cathepsin K) and *Acp5* (encoding tartrate-resistant acid phosphatase, TRAP) have been used to mark osteoclasts for *in vivo* studies. However, expression of these genes is not restricted to osteoclasts (https://www.immgen.org), and off-target expression may confound analysis (Han et al., 2019; Winkeler et al., 2012). This led us to develop an alternative high-fidelity system for marking osteoclasts *in vivo* using their physical and functional properties. We generated mixed bone marrow chimeras in which lethally irradiated mice were reconstituted with a 1:1 or 1:4 mix of bone marrow cells from separate donors where the cells singly express either a green or red fluorescent protein (Figure 1A). In this system, osteoclasts that form from the fusion of monocyte/macrophage lineage cells from the two donor lines are double-labeled red and green. We used *Lysm*^Cre/+^.*Tdtomato*^LSL/LSL^ mice (in which myeloid lineage cells expressing LYSM are red due to expression of tdTomato) and either *Blimp-1*^Egfp/+^.*Rag-1*^−/−^ mice (in which osteoclasts and natural killer cells are green due to expression of GFP) or *Csf1r*^Egfp/+^ mice (in which CSF1R^+^ cells are green due to expression of GFP) as donor bone marrow (Figure 1A). Osteoclasts were identified as large LYSM^+^BLIMP1^+^ or LYSM^+^CSF1R^+^ cells located adjacent to the endosteal bone surface in mosaic-tiled images of the tibia of recipient mice (Figure 1B; Video S1). These LYSM^+^CSF1R^+^ double-labeled cells were multinucleated and expressed cathepsin K, a key enzyme expressed by osteoclasts, and >90% had resorbed fluorescently labeled bisphosphonate from bone, confirming they were osteoclasts (Figures 1C–1E). While these cells shared similarities with *in vitro* generated osteoclasts (Figure 1F) (Arnett and Dempster, 1986; Boyce, 2013; Vesely et al., 1992), they were exceptional for their stellate appearance with multiple cellular processes contacting neighboring cells to form an interconnected network across the endosteum (Figure 1B). Although we cannot exclude the presence of migrating cells in the network, “branching” osteoclasts have been described in glutaraldehyde-fixed specimens (Jones and Boyde, 1977), where the cellular architecture is preserved prior to tissue processing. Intravital imaging over 2 h revealed remarkable stability in the size, dendritic morphology, and localization of these osteoclasts in the steady state (Figures 1G–1I; Video S2). Thus, osteoclasts *in vivo* differ from osteoclasts generated *in vitro* in their stellate appearance and extensive cell-to-cell contacts.

Video S7. Intravital imaging of osteoclast fission and cellular recycling, related to Figures 4A–4C Video shows neighboring stellate LYSM^+^ (red) BLIMP1^+^ (green) osteoclasts undergoing sRANKL-stimulated cell fission into multiple daughter cells and fusion of daughter cells with neighboring osteoclasts. These events are highlighted the cell fate mapping sequence. Time stamp is hh:mm:ss.

Video S8. Intravital imaging of osteoclast dynamics following OPG:Fc treatment and withdrawal, related to Figures 4D–4F and 5A–5D Initial part of the video shows the accumulation of numerous small rounded LYSM+ (red) CSF1R+ (green) osteomorphs from a mouse treated with OPG:Fc. Time stamp is hh:mm:ss. The next part of the video, in contrast, shows osteoclast recycling 3 weeks after OPG:Fc withdrawal. Osteoclasts are large and undergo iterative cycles of cell fission and fusion, as shown by the cell fate mapping. Time stamp is hh:mm:ss.

### Osteoclasts undergo cellular fusion *in vivo*

**Figure 2:**
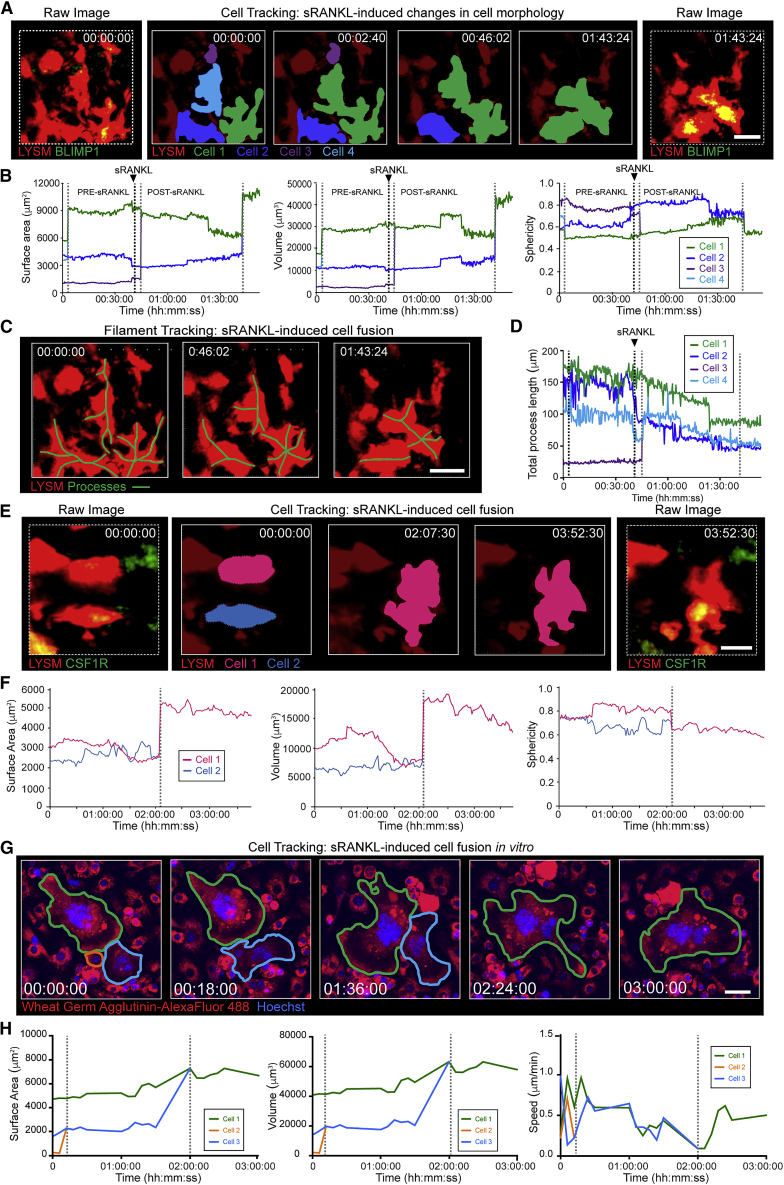
Intravital imaging of sRANKL-stimulated osteoclast dynamics and cell fusion (A) Cell tracking post-sRANKL stimulation. Raw images (left and right) show LYSM^+^ (red) and BLIMP1^+^ (green) expression. Timelapse (middle panels) shows tracked double-labeled osteoclasts. Scale bar, 30 μm. Timestamp hh:mm:ss. (B) Surface area, volume, and sphericity tracked in (A). (C) Tracking morphology of osteoclast from (A) using FilamentTracer. Cell processes shown by green lines. Scale bar, 30 μm. (D) Total process length in cells from (C). See also Video S3. (E) Cell tracking post-sRANKL stimulation. Raw images (left and right) show LYSM^+^ (red) and CSF1R^+^ (green) expression. Scale bar, 20 μm. Time-stamp hh:mm:ss. (F) Surface area, volume, and sphericity of cells tracked in (E). See also Video S4. (G) Live-cell imaging of osteoclast fusion post-sRANKL stimulation *in vitro*. Cells labeled with wheat germ agglutinin-AlexaFluor 488 (red pseudocolor) and Hoechst (cyan). Scale bar, 40 μm. Timestamp hh:mm:ss. See also Video S4. (H) Surface area, volume, and speed of cells tracked in (G).

Since the RANKL/RANK/OPG signaling system is the physiological master regulator of osteoclast biology and bone resorption, we chose to manipulate this system to determine its impact on osteoclast dynamics. Soluble RANKL-stimulated osteoclasts were highly dynamic and underwent morphological changes, which began within 20 min of sRANKL administration by retracting their cellular processes (Figures 2A–2D; Video S3). In this activated state, osteoclasts migrated toward each other and appeared to undergo cell fusion to form larger cells (Figure 2E and 2F; Videos S3 and S4). A limitation of two-photon imaging through intact cortical bone is the need to trade off the detection of weak signals from fine cellular processes with the saturation of strong signals from cell bodies. Therefore, to confirm that the cells do fuse, we performed *in vitro* live cell imaging by confocal microscopy. This also showed the fusion of multinucleated osteoclasts to form even larger polykaryons (Figures 2G and 2H; Video S4; Jansen et al., 2012). Thus, sRANKL-stimulated osteoclasts are larger and more active than steady-state osteoclasts, both *in vivo* and *in vitro*.

Video S1. Osteoclasts are large stellate cells that form an interconnected network, related to Figure 1B Video shows mosaic tiled z stack images of tibia showing the endosteal bone (blue; second harmonic generation, SHG) and network of interconnecting large stellate cells expressing LYSM (red) and CSF1R (green). These z stacks were rendered in 3D to show bone surface and cells located on the endosteum that express LYSM and CSF1R. Final sequence show the 3D volume render and cells that co-localize LYSM and CSF1R.

Video S2. Intravital imaging of steady-state osteoclast dynamics, related to Figures 1G and 1H Video shows a stellate LYSM^+^ (red) CSF1R^+^ (green) osteoclast with multiple cellular processes contacting neighboring cells. For clarity, the red channel only is then shown followed by tracking of the cell processes (yellow) using FilamentTracer. Time stamp is hh:mm:ss.

### Osteoclasts undergo cellular fission *in vivo*

**Figure 3:**
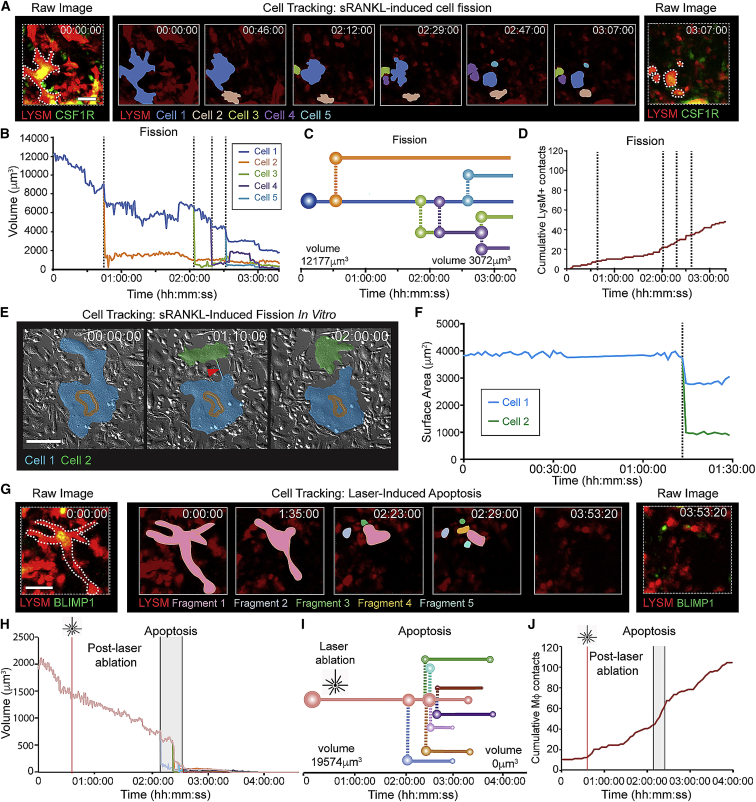
Intravital imaging of sRANKL-stimulated osteoclast cell fission (A) Cell tracking showing osteoclast fission. Raw images (left and right) show LYSM^+^ (red) and CSF1R^+^ (green) expression. Timelapse (middle panels) shows tracked double-labeled osteoclast. Scale bar, 25 μm. Timestamp hh:mm:ss. See also Video S5. (B) Cell volume from (A). (C) Cell fate mapping from (A) showing fission of parent cell into five daughter cells. (D) Cumulative number of contacts between LYSM^+^CSF1R^neg^ macrophages and fission products. Dotted lines are fission events. (E) Live-cell imaging of cell fission *in vitro*. Scale bar, 40 μm. Timestamp is hh:mm:ss. (F) Surface area from (E). See also Video S5. (G) Cell tracking showing cell undergoing laser-induced apoptosis. Raw images (left and right) show LYSM^+^ (red) and BLIMP1^+^ (green) expression. Timelapse (middle panels) shows tracked double-labeled osteoclast and apoptotic fragments. Scale bar, 40 μm. Timestamp hh:mm:ss. See also Video S6. (H) Cell volume from (G). Red line identifies laser ablation. Grey window identifies apoptotic events. (I) Cell fate mapping of apoptotic cell fragments in (G). Circles represent relative cell volume on a log_10_ scale. (J) Cumulative contacts between LYSM^+^CSF1R^neg^ macrophages and apoptotic cell fragments. Red line identifies laser ablation.

In long-term intravital imaging experiments, we also observed that large sRANKL-stimulated osteoclasts often fissioned into smaller daughter cells (Figures 3A–3C; Video S5). These cells were larger than 5 μm in diameter and freely motile, suggesting that they were live. Analysis of time-lapse images confirmed they represented true cell fission events (Video S5). Fission was not accompanied by changes to the level of interaction with macrophages (Figure 3D). *In vitro* live-cell imaging showed similar cell fission events, and these nucleated daughter cells were motile (Figures 3E and 3F; Video S5).

Video S3. Intravital imaging of sRANKL-stimulated osteoclast dynamics, related to Figures 2A–2D Video shows neighboring stellate LYSM^+^ (red) BLIMP1^+^ (green) osteoclasts before and after administration of sRANKL. Initial maximal intensity projection sequence shows the osteoclasts retract their processes, migrate toward each other and undergo cell-to-cell fusion. The next sequence shows the cell fate mapping and tracking of the processes (yellow) using FilamentTracer. The final sequence show a single z stack and crop and 3D rotation of the fused cell. Time stamp is hh:mm:ss.

### Osteoclasts fission is distinct from apoptosis

We next sought to exclude the possibility that this represented apoptosis. We did not detect spontaneous apoptosis in >130 h of intravital imaging, so we developed a system to induce osteoclast apoptosis *in vivo*. The bisphosphonate clodronate is reported to induce osteoclast apoptosis *in vitro* and *in vivo* (Hughes et al., 1995; Selander et al., 1996); however, we could not detect apoptosis following clodronate treatment. We therefore used localized delivery of two-photon laser pulses (Chtanova et al., 2014) to induce osteoclast apoptosis *in vivo* by phototoxicity (Figure 3G; Video S6). Irradiation of osteoclasts resulted in fragmentation of the cell into multiple smaller subcellular components <5 μm in diameter. These fragments were rounded, non-motile, and eventually disappeared, possibly due to engulfment and digestion by macrophages. Thus, while the total volume of daughter cells was preserved and similar to the volume of the parental cell in osteoclast fission (Figure 3H), in apoptosis the total volume of fragments decreased to zero (Figure 3I). Moreover, while osteoclast fission was immunologically “silent’” (Figures 3A and 3D; Video S5), apoptosis was accompanied by an influx of monocytes/macrophages, which scanned and interacted with debris at a higher rate than in cell fission (Figures 3G and 3J; Video S6). These data establish that *in vivo*, in response to sRANKL, osteoclasts undergo fission to produce daughter cells in a process that is distinct from apoptosis.

Video S4. Intravital imaging of RANKL-stimulated osteoclast cell fusion, related to Figures 2E–2H Video shows neighboring stellate LYSM^+^ (red) BLIMP1^+^ (green) osteoclasts undergoing sRANKL-stimulated cell fusion. Initial maximal intensity projection sequence shows the osteoclasts migrate toward each other and undergo cell-to-cell fusion. The next sequence shows the cell fate mapping and tracking of the processes (yellow) using FilamentTracer followed by a sequence showing a single z stack and crop and 3D rotation of the fused cell. Time stamp is hh:mm:ss. The final sequence shows live cell imaging of osteoclast cell fusion *in vitro* with sRANKL-treated multinucleated osteoclasts labeled with Wheat Germ Agglutinin-AlexFluor 488 (pseudocolor red) and Hoechst (blue) migrating and undergoing cell-to-cell fusion in culture. Time stamp is hh:mm:ss.

### Osteoclasts undergo cellular recycling *in vivo*

**Figure 4:**
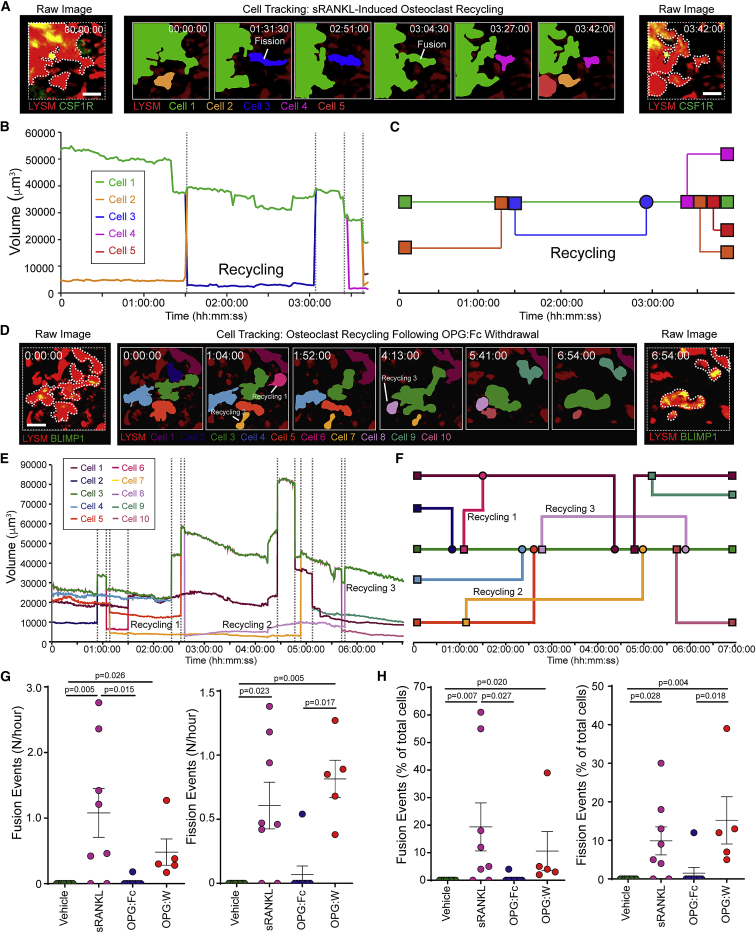
Osteoclasts recycle via osteomorphs (A) Cell tracking showing osteoclast recycling following sRANKL stimulation. Raw images (left and right) show LYSM^+^ (red) and CSF1R^+^ (green) expression. Timelapse (middle panels) shows tracked double-labeled osteoclast undergoing fission and fusion. Scale bar, 40μm. Timestamp hh:mm:ss. (B) Cell volume from (A). (C) Cell fate mapping from (A) showing a cell recycling (blue). See also Video S7. (D) Cell tracking showing osteoclast recycling following withdrawal of OPG:Fc treatment. Raw images (left and right) show LYSM^+^ (red) and BLIMP1^+^ (green) expression. Timelapse (middle panels) shows tracked double-labeled osteoclast undergoing recycling. Scale bar, 50 μm. Timestamp hh:mm:ss. (E) Cell volume from (D). (F) Cell fate mapping from (D) showing multiple fission, fusion, and cell recycling events. See also Video S8. (G) Number of cell fusion events and cell fission events in mice treated with vehicle (n = 8), sRANKL (n = 7), OPG:Fc (n = 8), or following withdrawal of OPG:Fc treatment (OPG:W) (n = 5). Each data point represents one mouse. Mean ± SEM are shown. (H) Percent of cells (undergoing fusion events and fission events in mice treated with vehicle, sRANKL, OPG:Fc, or following OPG:W). Each data point represents one mouse. Mean ± SEM are shown. See also Figure S1.

**Figure S1:**
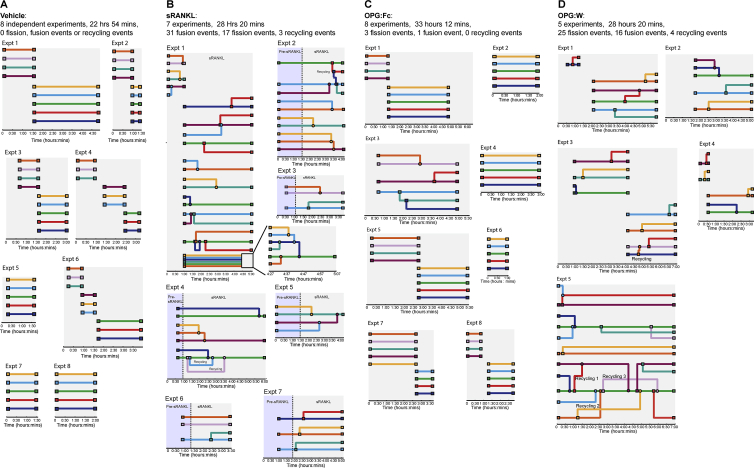
“Underground” cell fate maps depicting the fate of individual cells, related to Figures 3 and 4 (A) Underground maps of cells from mice treated with vehicle. (B) Underground maps of cells from mice treated with sRANKL. (C) Underground maps of cells from mice treated with OPG:Fc. (D) Underground maps of cells from mice following OPG:Fc withdrawal (OPG:W). Nodes represent cells and intersecting lines indicate fission or fusion events. Different colors represent different cells. Each map represents a replicate experiment. The number of experiments, the duration of imaging, the number of fission, fusion and recycling events and the number of events per hour are indicated.

We next sought to determine the fate of the daughter cells. Tracking showed they not only migrated away from the parent polykaryon, but they also fused with neighboring osteoclasts and in some cases with each other (Figures 4A–4C; Video S7). Thus, daughter cells were “recycled” into larger polykaryons. This cellular recycling was also observed *in vitro*. Recycling events were visualized by cell fate mapping trees (Figure 4C; Figure S1) and fission and fusion of recycling cells lead us to name them “osteomorphs” to distinguish them from osteoclasts.

Video S5. Intravital imaging of sRANKL-stimulated osteoclast cell fission, related to Figures 3A–3F Video shows neighboring stellate LYSM^+^ (red) BLIMP1^+^ (green) osteoclasts undergoing sRANKL-stimulated cell fission. Initial maximal intensity projection sequence shows the osteoclasts breaking up into multiple smaller motile cells that migrate away from each other. The next sequence shows the cell fate mapping. Time stamp is hh:mm:ss. The final sequence shows live cell imaging of osteoclast cell fission *in vitro* with an osteoclast undergoing fission into two large daughter cells. Red arrow highlights the retraction nanotube. Time stamp is hh:mm:ss.

### Osteoclast recycling is regulated by RANKL signaling

Since sRANKL induced osteoclast recycling, we next determined the impact of therapeutic inhibition on this process. This was achieved by administering OPG:Fc, the decoy receptor for RANKL, which inhibits RANK signaling, and then withdrawing it (OPG:W). This was compared to mice treated with vehicle, sRANKL, or OPG:Fc alone. The number of fission, fusion, and recycling events were quantified and visualized using cell fate maps (Figure S1). Osteoclast recycling was not observed in the steady state (Figures 1G–1I; Figure S1; Video S2). Mice treated with sRANKL underwent cycles of fission and fusion when imaged for up to 7 h (Figures 4A–4F; Video S7). Treatment with OPG:Fc inhibited osteoclast recycling (Figure S1; Video S8). However, 3 weeks after OPG:Fc was withdrawn, osteoclasts underwent cycles of fission and fusion similar to that seen in mice treated with sRANKL (Figures 4D–4F; Figure S1; Video S8). Analysis of the frequency of events showed that in the absence of treatment, fusion and fission was not detected in the 7 h analysis timeframe. In contrast, sRANKL treatment was associated with 1.08 ± 0.37 fusion and 0.61 ± 0.18 fission events/h. This was inhibited with OPG:Fc (fusion 0.02 ± 0.02/h, fission 0.07 ± 0.07/h) but increased following OPG:Fc withdrawal (fusion 0.48 ± 0.20/h, fission 0.81 ± 0.15/h) (Figure 4G). In this timeframe, 19.38% ± 8.71% of cells underwent cell fusion and 9.88% ± 3.62% of cells underwent cell fission events in sRANKL-treated mice, suggesting this is a common phenomenon (Figure 4H). OPG:Fc treatment reduced the cells undergoing fusion and fission to untreated control levels (fusion 0.44% ± 0.44%, fission 1.33% ± 1.33%). Strikingly, OPG:W increased the proportions of cells undergoing fusion and fission to proportions seen with sRANKL treatment (fusion 10.60% ± 7.12%, fission 15.20% ± 6.14%) (Figure 4H; Figure S1). Thus, osteoclast recycling is regulated by RANKL signaling *in vivo*.

Video S6. Intravital imaging of osteoclast apoptosis, related to Figures 3G–3J Apoptosis was induced by localized two-photon photoablation using a near-infrared laser. White box highlights a LYSM^+^ (red) BLIMP1^+^ (green) osteoclast fragmenting into small non-motile subcellular components and recruitment of LYSM^+^ macrophages to scan and clear the debris. Time stamp is hh:mm:ss.

### RANKL inhibition results in the accumulation of osteomorphs

**Figure 5:**
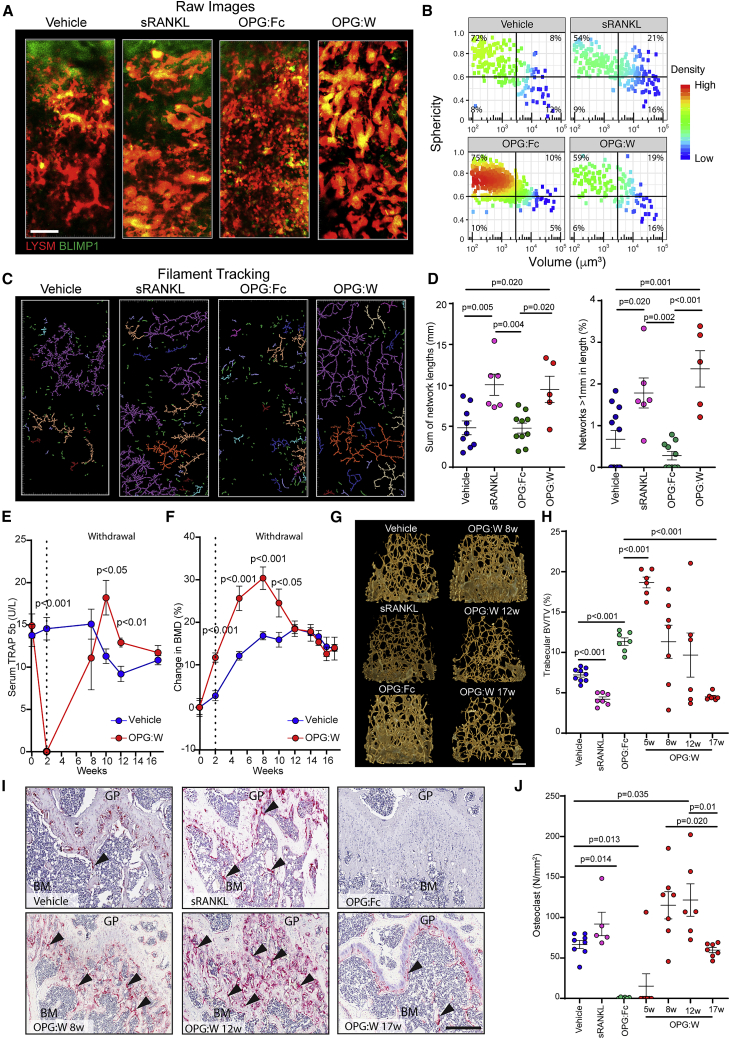
Regulation of osteoclast recycling by RANKL signaling (A) Raw images of LYSM^+^BLIMP1^+^ osteoclasts from mice treated with vehicle (n = 9), sRANKL (n = 6), OPG:Fc (n = 8), or following OPG:W (n = 6). Scale bar, 60 μm. (B) Pseudocolor density plot of volume and sphericity for double-labeled cells that have taken up fluorescent bisphosphonate analyzed in (A). (C) Network analysis using FilamentTracer of cells in (A). Percentage of cells in each quadrant are shown. (D) Sum of network lengths and the proportion of networks >1 mm in length from (C). Each data point represents one mouse. Mean ± SEM are shown. (E) Longitudinal changes in serum TRAP 5b in mice treated with vehicle (n = 9) and following OPG:W (n = 8). Mean ± SEM are shown. (F) Longitudinal changes in BMD in mice treated with vehicle and OPG:W. Mean ± SEM are shown. (G) Micro-computed tomography (CT) images of cancellous bone in the femur of mice treated with vehicle (n = 9), sRANKL (n = 7), OPG:Fc (n = 7), and OPG:W at 5 (n = 6), 8 (n = 8), 12 (n = 6), and 17 (n = 7) weeks (w). Scale bar, 200 μm. (H) Trabecular bone volume/tissue volume (BV/TV) calculated from (G). (I) Histological images of the femur stained for TRAP (red) showing osteoclasts (arrow heads) in mice treated with vehicle (n = 8), sRANKL (n = 5), OPG:Fc (n = 6), and OPG:W at 5 (n = 7), 8 (n = 7), 12 (n = 6), and 17 (n = 7) w. BM, bone marrow; GP, growth plate. Scale bar, 50 μm. (J) Number of TRAP^+^ osteoclasts per mm of bone surface. Each data point represents one mouse. Mean ± SEM are shown. See also Figure S2.

Since RANKL regulates osteoclast recycling, we sought to quantify the effect of sRANKL, OPG:Fc, and OPG:W on the number, morphology, and network connections between double-labeled cells that have taken up bisphosphonate (Figures 5A–5D). OPG:Fc treatment induced the loss of large aspherical cells >10,000 μm^3^ and a shift in the distribution of the cell volume and sphericity, such that overall cells were smaller and more rounded (Figures 5A and 5B). Network analysis showed that these cells did not form the extensive interconnected networks of cells >1,000 μm in length seen in steady-state conditions and in sRANKL-treated mice (Figures 5C and 5D). This suggests that OPG:Fc inhibits RANKL-dependent fusion, resulting in the accumulation of osteomorphs.

**Figure S2:**
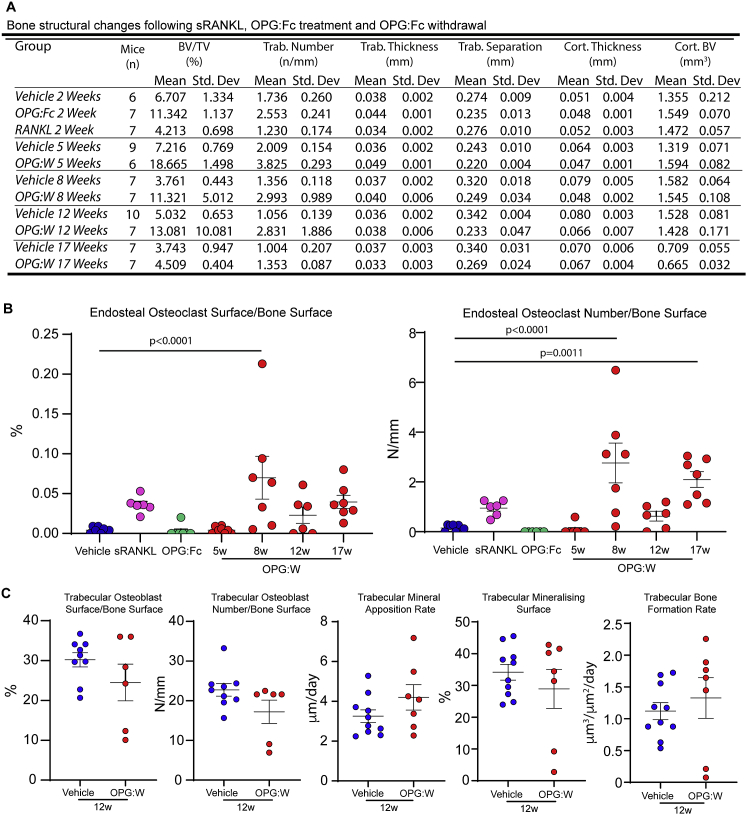
Bone structure and activity in mice treated with vehicle, sRANKL, OPG:Fc, and following OPG:Fc withdrawal, related to Figure 5 (A) Micro-CT data showing effect on bone microarchitecture. (B) Enumeration of osteoclast cell surface (left) and numbers (right) per unit bone surface. Vehicle (n = 8), sRANKL (n = 5), OPG:Fc (n = 6) and OPG:W at 5 (n = 7), 8 (n = 7), 12 (n = 6) and 17 (n = 7) weeks (w). (C) Histomorphometry showing effect on osteoblast cell surface (far left) and number (left), osteoblast activity (middle, right, far right panels). Vehicle (n = 9-10) and OPG:W (n = 6-7).

To determine the consequence of this osteomorph accumulation, we longitudinally analyzed mice treated with OPG:Fc for 2 weeks and then for a further 15 weeks after OPG:Fc withdrawal. Serum TRAP 5b was suppressed during OPG:Fc treatment and rebounded upon OPG:W (Figure 5E). This was matched by an initial increase, peaking at 8 weeks, and subsequent decrease in BMD (Figure 5F). These changes were paralleled by changes in bone microarchitecture (Figures 5G and 5H; Figure S2). OPG:Fc treatment was associated with a significant decrease in TRAP^+^ osteoclasts, and cessation of OPG:Fc treatment was followed by a corresponding rebound increase in TRAP+ osteoclasts (Figures 5I and 5J). These data suggest that accumulation of osteomorphs in a recycling pool may contribute to the accelerated bone loss and pathological vertebral fractures observed in a subset of patients who have discontinued denosumab therapy (Anastasilakis et al., 2017a; 2017b; McClung et al., 2017; Popp et al., 2016; Tsourdi et al., 2017; Zanchetta et al., 2018).

### Osteomorphs fuse to form functional osteoclasts

**Figure 6:**
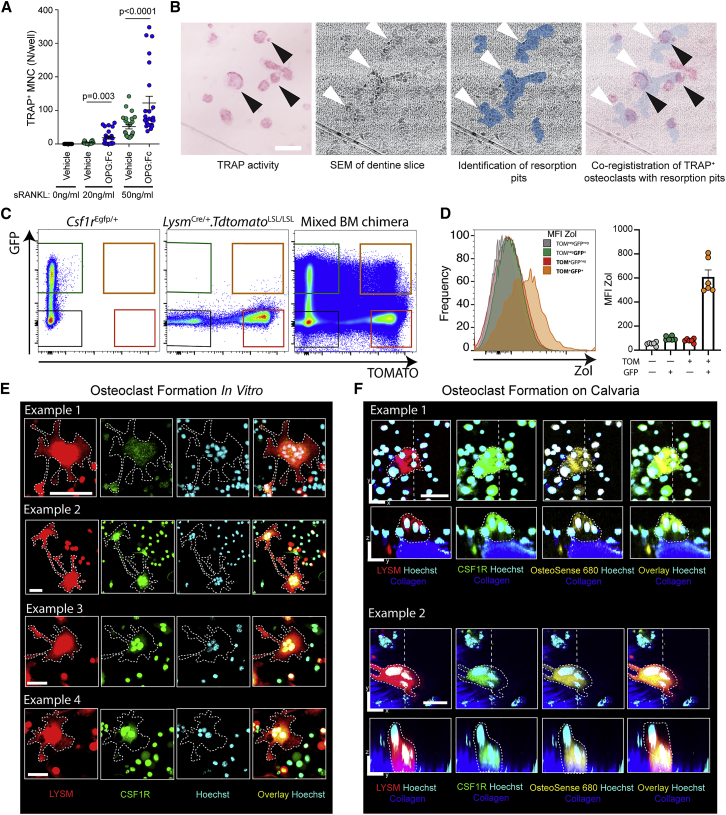
Osteomorphs reassemble into osteoclasts capable of resorbing bone (A) Number of TRAP^+^ multinucleated cells generated from bone marrow mononuclear cells isolated from mice treated with vehicle (n = 2) or OPG:Fc (n = 2) and cultured in the presence of increasing doses of sRANKL. Mean ± SEM are shown. (B) Cells isolated from mice in (A) directly seeded onto dentine slices and cultured in the presence of M-CSF and sRANKL. TRAP^+^ osteoclasts (black arrowheads) and resorption pits on dentine slices (white arrowheads) as shown by scanning electron microscopy (SEM). Scale bar, 100 μm. (C) FACS analysis showing LYSM and CSF1R expression in *Lysm*^Cre/+^.*Tdtomato*^LSL/LSL^, *Csf1r*^Egfp/+^, and *Lysm*^Cre/+^.*Tdtomato*^LSL/LSL^:*Csf1r*^Egfp/+^ mixed bone marrow chimeras. (D) Histogram (left panel) showing uptake of zoledronic acid (ZOL) by cells in the indicated gates from *Lysm*^Cre/+^.*Tdtomato*^LSL/LSL^:*Csf1r*^Egfp/+^ mixed bone marrow chimeras in (C). Right panel shows MFI for ZOL for the identified cell populations for individual mice (n = 6). Mean ± SEM are shown. (E) FACS-sorted LYSM^+^CSF1R^+^ cells cultured with non-fluorescent osteoclasts *in vitro* (four examples). Individual LYSM^+^ (red), CSF1R^+^ (green), and Hoechst (cyan) channels and the overlay are shown. Dotted lines identify osteoclasts. Scale bars, 50 μm. (F) FACS-sorted LYSM^+^CSF1R^+^ cells cultured on isolated calvarial bone (two examples). LYSM (red), CSF1R (green), and OsteoSense 680 (yellow) channels with Hoechst (cyan) and the overlay are shown. Dotted lines identify osteoclasts. Note nuclei of endogenous non-fluorescent cells. Scale bar, 20 μm.

**Figure S3:**
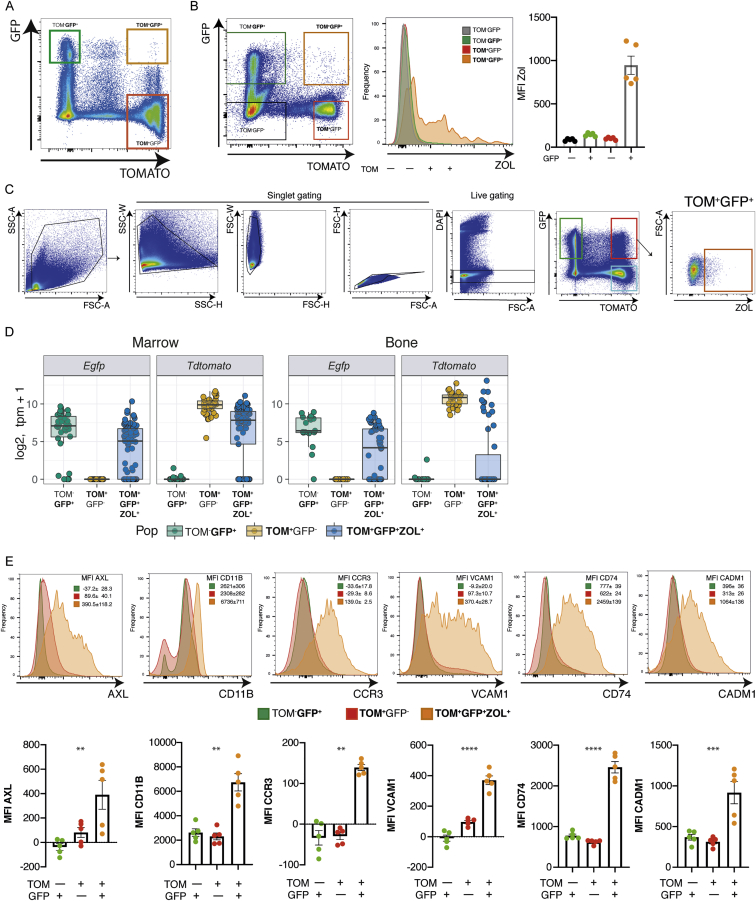
Identification and isolation of triple-positive putative osteomorphs from marrow and osteoclasts from bone by FACS analysis and cell sorting, related to Figures 6 and 7 (A) Pseudocolor density plot shows the presence of circulating TOM^+^GFP^+^ cells in the blood in mixed bone marrow chimeras. (B) Pseudocolor density plot (left panel) shows the presence of TOM^+^GFP^+^ cells in the bone compartment in mixed bone marrow chimeras. Histogram overlay (middle panel) shows double-labeled cells selectively take up fluorescent bisphosphonate and are also ZOL^+^. Graph of ZOL MFI (right panel) for each of the indicated populations. (C) Gating strategy for FACS sorting of single-positive and triple-positive cells from marrow and bone. Pseudocolor density plot of single cell suspension from marrow showing single cell gates for doublet exclusion, gating for red and green single-positive cells (green box) and double-labeled cells that are also ZOL^+^ (red box). (D) Expression of the *Egfp* and *Tdtomato* genes in single cells sorted from marrow and bone for scRNA-seq. (E) FACS analysis comparing the expression of a number of osteomorph markers on single- and triple-positive cells from the marrow. Overlay histograms (top panels) show AXL, CD11B, CCR3, VCAM1, CD74 and CADM1 with mean ± SEM of the respective MFIs. Individual data points are plotted in bottom panels.

OPG:Fc withdrawal resulted in an increase in osteoclastic activity with increased numbers of large polykaryons >10,000 μm^3^ with low sphericity (Figures 5A and 5B) and a corresponding increase in the number of networks and total networks with lengths >1,000 μm (Figures 5C and 5D). These changes resembled those observed in mice treated with sRANKL and suggested that OPG:Fc treatment increased the reservoir of osteomorphs that are poised to fuse and reassemble into bone resorbing osteoclasts upon drug withdrawal. To test this, we isolated bone marrow cells from OPG:Fc-treated mice and cultured them *in vitro* with M-CSF and sRANKL (Figure 6A). The same number of bone marrow cells isolated from OPG:Fc-treated mice generated more TRAP^+^ osteoclasts than cells from vehicle-treated mice. These TRAP^+^ cells were able to form resorption pits on dentine slices (Figure 6B). To show that this was due to the increase in osteomorphs, we set out to identify cells that co-expressed green and red fluorescence from the bone marrow of chimeric mice by fluorescence-activated cell sorting (FACS) (Figure 6C). Since osteoclasts generated from the fusion of green and red cells were double labeled, we reasoned that osteomorphs generated by the fission of these osteoclasts would retain expression of fluorescent proteins and also be double labeled. Furthermore, since osteomorphs were motile, we reasoned that they could be recovered from the bone marrow. Consistent with this notion, double-labeled cells can be detected in the blood of mixed chimeras, indicating that they are able to detach from bone and recirculate (Figure S3). Analysis of the mean fluorescence intensity (MFI) showed that double-labeled cells were zoledronic acid positive (ZOL^+^), suggesting they were derived from osteoclasts that had resorbed bone and taken up bisphosphonate bound to the bone surface (Figure 6D). We next sorted these LYSM^+^CSF1R^+^ (TOM^+^GFP^+^) putative osteomorphs using stringent singlet gates to exclude doublets and dead cells (Figure S3). Double-labeled cells were sorted, co-cultured unlabeled osteoclasts (Figure 6E), and also seeded onto the calvarial bone for *ex vivo* culture (Figure 6F). These experiments showed that putative osteomorphs were able to undergo cell fusion and form multinucleated cells that resorb bone and take up bisphosphonate. Thus, osteomorphs derived from osteoclast fission are able to fuse into mature bone resorbing osteoclasts.

### Osteomorphs are distinct from osteoclasts and macrophages

**Figure 7:**
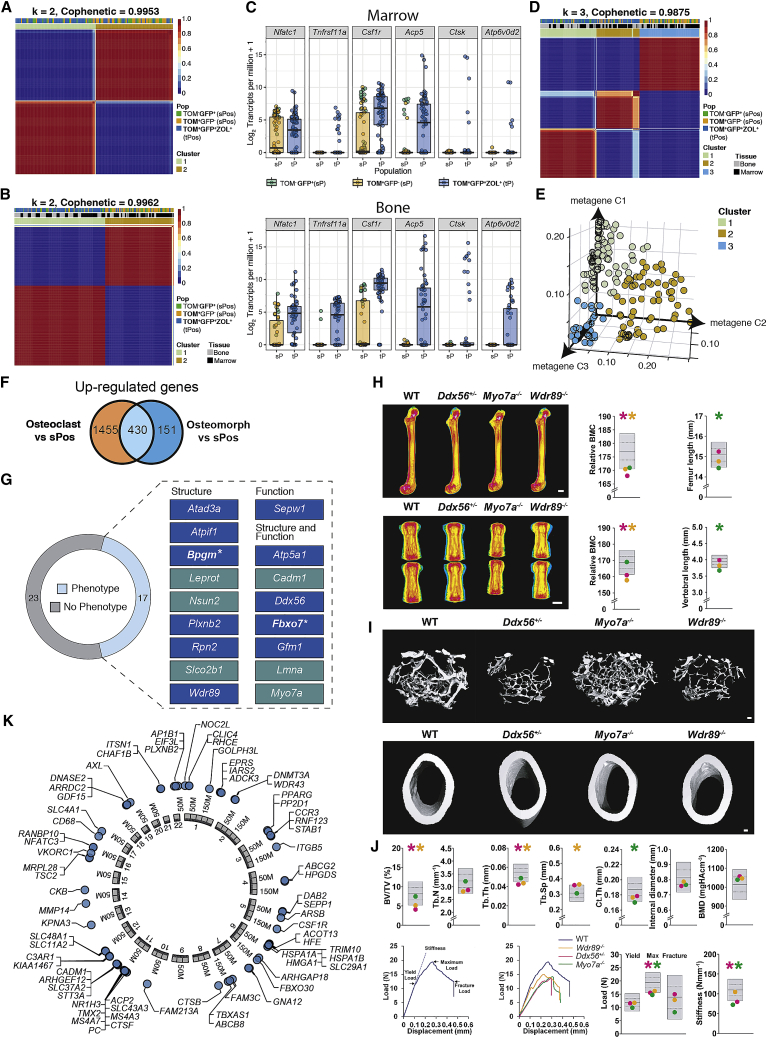
Osteomorph genes control bone structure, function, and disease LYSM^neg^CSF1R^+^ (TOM^neg^GFP^+^) and LYSM^+^CSF1R^neg^ (TOM^+^GFP^neg^) single-positive cells (sPos) and LYSM^+^CSF1R^+^Zol^+^ (GFP^+^TOM^+^ZOL^+^) triple-positive (tPos) cells were index sorted from marrow and bone by FACS and analyzed by scRNA-seq. (A) Consensus plot showing cells in the marrow fraction decomposed into two clusters made up predominantly of tPos cells in one group (cluster 1) and GFP^+^ and TOM^+^ sPos (cluster 2) in another. (B) Consensus plot showing cells in both the marrow and bone fraction can be decomposed into two made up predominantly of tPos cells in one group (cluster 1) and sPos (cluster 2) in another irrespective of their tissue compartment. (C) Expression of canonical osteoclast genes by sPos and tPos cells isolated from bone marrow (top) and the bone fraction (bottom). (D) Consensus plot showing the marrow and bone fraction can be further decomposed into three clusters made up of tPos cells from bone (cluster 1), tPos cells from marrow (cluster 2), and sPos cells from both marrow and bone (cluster 3). (E) Visualization of three cell clusters based on their metagene expression. (F) Venn diagram showing overlap of genes upregulated in tPos cells isolated from bone (enriched with osteoclasts) and marrow (enriched with osteomorphs) compared to sPos macrophages isolated from the bone and marrow. (G) Genes upregulated in osteomorphs, with (light blue) and without (gray) outlier bone phenotypes, in 40 knockout mouse lines from the OBCD database. Eleven genes (blue box) have not been previously reported to be involved in skeletal structure or function. Two genes (asterisks) were uniquely upregulated in osteomorphs but not osteoclasts. (H) Representative quantitative X-ray microradiographic images of the femurs (top) and vertebrae (bottom) of adult, female wild-type (WT), *Ddx56*^+/−^, *Myo7a*^−/−^, and *Wdr89*^*−/−*^ mice. Scale bar, 1 mm. Dot plots illustrate summary data for individual parameters. For each variable, the mean (center line), ± 1 SD (middle lines), and ± 2 SD (gray boxes) for WT mice (n = 320) are shown. Mean values (colored dots) for each parameter in *Ddx56*^+/−^ (orange), *Myo7a*^−/−^ (green), and *Wdr89*^*−/−*^ (pink) mouse lines are shown. Genes were considered outliers if the mean was >2 SD from the WT reference mean and are denoted by an asterisk (^∗^) and colored according to the individual mouse line. (I) Representative micro-CT images of cancellous (top) and cortical (bottom) bone of adult, female WT, *Ddx56*^+/−^, *Myo7a*^−/−^, and *Wdr89*^*−/−*^ mice. Scale bar, 100 μm. Dot plots illustrate summary data for bone volume as a proportion of tissue volume (BV/TV), trabecular number (Tb.N), trabecular thickness (Tb.Th), trabecular separation (Tb.Sp), cortical thickness (Ct.Th), internal endosteal diameter, and bone mineral density (BMD). Data are described, and outlier phenotypes identified, as in (H). (J) Model load displacement curves (left panel) and summary displacement curves (right panel) from three-point bend testing of the femur of adult, female WT, *Ddx56*^+/−^, *Myo7a*^−/−^, and *Wdr89*^*−/−*^ mice. Dot plots illustrate summary data for yield load, maximum load, fracture load, and stiffness. Data are described, and outlier phenotypes identified, as in (H). (K) 71 of the 520 osteomorph upregulated genes with human orthologs are significantly (P_MULTI_ < 2.4E-6) associated with eBMD in the UK Biobank study. Chromosomes number and genome coordinates of each human ortholog are shown.

To cross-validate the existence of osteomorphs and identify the genes that define the osteomorph cell state, we performed scRNA-seq. We hypothesized that single-positive LYSM^+^CSF1R^neg^ and LYSM^neg^CSF1R^+^ are monocyte/macrophage precursors, triple-positive LYSM^+^CSF1R^+^ZOL^+^ cells from the bone marrow are osteomorphs, and triple-positive cells from the bone surface are osteoclasts (Figure S3). We first analyzed the cells from marrow to determine if triple-positive osteomorphs were distinct from the single-positive cells. Analysis of *Egfp* and *Tdtomato* gene expression showed that green cells expressed *Egfp* but not *Tdtomato*, red cells expressed *Tdtomato* but not *Egfp*, and triple-positive cells expressed both fluorescent reporters (Figure S3). Unsupervised dimensionality reduction of differentially expressed genes by non-negative matrix factorization (NMF) revealed that red-only and green-only single-positive cells clustered together and were distinct from triple-positive cells (with a cophenetic coefficient for k = 2 of 0.9953, indicating near-perfect clustering of the data into two groups) (Figure 7A). Similarly, when all six sorted cell populations were analyzed, single-positive cells clustered together away from triple-positive cells, regardless of whether they were positioned in the marrow or bone surface (with a cophenetic coefficient for k = 2 of 0.9962, indicating near-perfect clustering into two groups) (Figure 7B). Analysis of canonical osteoclast genes (*Nfatc1*, *Tnfrsf11a*, *Csf1r*, *Acp5*, *Ctsk*, and *Atp6v0d2*) revealed that they were expressed by triple-positive, but not single-positive, cells from both sites (Figure 7C). These data suggest that triple-positive cells from bone are osteoclasts and related to triple-positive cells from the marrow.

To discover hidden classes of cells within the dataset, we next asked if a model in which k = 3 also fitted the data. This analysis showed that triple-positive cells from bone (osteoclasts) clustered in one group, triple-positive cells from marrow (osteomorphs) clustered in a second group, and single-positive cells from both bone and marrow clustered together in a third group (with cophenetic coefficient for k = 3 of 0.9875, indicating extremely good clustering of the data into three groups) (Figure 7D). These three cell clusters can be visualized by their expression of metagenes 1, 2, and 3 (Figure 7E). Comparison of triple-positive marrow osteomorphs to single-positive macrophage precursors revealed 581 upregulated genes (Table S1). 430 of the 581 (74%) genes upregulated by triple-positive marrow osteomorphs were also upregulated by triple-positive osteoclasts, indicating overlap in their gene expression profile (Figure 7F; Table S1). On the other hand, these 430 genes comprised only 23% of the 1,885 genes upregulated by triple-positive bone osteoclasts compared to single-positive macrophage precursors, suggesting that expression of a large number of these genes has been downregulated in marrow osteomorphs (Figure 7F). Osteoclasts expressed higher levels of *Ctsk* and *Atp6v0d2* than osteomorphs (Figure 7C). Interestingly, 151 of the 581 (26%) genes upregulated by osteomorphs were not upregulated by osteoclasts (Figure 7F). This suggests that these two triple-positive cell populations, while similar on one level, are distinct on another. These data demonstrate that osteomorphs in the marrow, derived from the fission of osteoclasts on bone, are transcriptionally distinct from osteoclasts and macrophages.

To cross-validate the scRNA-seq data, we performed FACS analysis of a number of osteomorph upregulated genes that encode cell-surface proteins (Figure S3). This confirmed that AXL, CD11b, CCR3, VCAM1, CD74, and CADM1 are expressed at higher levels on the surface of osteomorphs than macrophages. These markers may therefore be useful to identify osteomorphs in non-chimeric mice and humans.

### Osteomorph genes control bone structure and function

To determine the role of osteomorph upregulated genes in bone biology, we screened mice with single gene deletions that have undergone skeletal phenotyping by our Origin of Bone and Cartilage Disease (OBCD) program (Freudenthal et al., 2016). Of the 581 upregulated osteomorph genes, detailed phenotyping was available for 40 mouse lines in which one or both copies of the gene had been deleted (Figure 7G; Table S2). Seventeen (42.5%) of these lines had structural and/or functional phenotypes. Six of the 17, cellular adhesion molecule 1 (*Cadm1*), lamin A/C (*Lmna*), leptin receptor gene-related protein (*Leprot*), myosin VIIA (*Myo7a*), NOP2/Sun RNA methyltransferase 2 (*Nsun2*), and solute carrier organic anion transporter family member 2B1 (*Slco2b1*) were in genes with an established role in the skeleton (Figure 7G; Bassett et al., 2012). The other 11 were in genes not previously annotated in the Gene Ontology (GO) or Mouse Genome Informatics (MGI) databases to have skeletal terms or bone phenotypes, suggesting they may be regulators of skeletal function. Two of the 11 genes (bisphosphoglycerate mutase, *Bpgm*, and F-box protein 7, *Fbxo7*) were significantly upregulated in osteomorphs but not in osteoclasts compared to macrophages. Structural and functional data for three examples of the 17 genes are shown: the DEAD-box helicase 56 *(Ddx56*), an ATP-dependent RNA helicase; *Myo7a*, a motor protein associated with Usher syndrome type 1B in humans (Weil et al., 1995); and the WD repeat domain 89 gene (*Wdr89*), a microtubule-associated protein involved in brain development and autophagy (Kannan et al., 2017) (Figures 7H–7J; Table S2). *Ddx56*^*+/−*^ mice had reduced femoral BMC, decreased trabecular bone volume (BV/TV), trabecular thickness (Tb.Th), reduced femoral maximum load and stiffness, and reduced vertebral BMC and stiffness (Figures 7H–7J; Table S2). *Myo7a*^*−/−*^ had reduced femoral length and cortical thickness and decreased maximum load and femoral stiffness, whereas, *Wdr89*^*−/−*^ had reduced femoral and vertebral BMC, femoral structural parameters, including BV/TV, Tb.Th, and trabecular separation but not functional parameters (Figures 7H–7J; Table S2). Detailed skeletal phenotyping for the other 14 mouse lines are documented in Table S2. These data indicate that osteomorph upregulated genes play an important structural and functional role and include genes not previously known to affect the skeleton.

### Osteomorph genes are involved in human skeletal diseases

Analysis of 520 human orthologs of genes that define osteomorphs in the *ISDS Nosology and Classification of Skeletal Disorders* database (Mortier et al., 2019) showed that 22 osteomorph genes cause monogenic skeletal dysplasia (1.5-fold enrichment, p = 0.03) (Table S3). Osteomorph genes were not uniformly involved but enriched in specific groups including the “Osteopetrosis and related disorders group” (5.2-fold enrichment, p = 0.02), “Lysosomal storage diseases with skeletal involvement group” (5.3-fold, p = 2 × 10^−3^), and the “Osteolysis group” (10-fold, p = 3 × 10^−3^) (Table S3).

To determine whether variants in human orthologs of osteomorph genes were enriched for genes associated with bone homeostasis in humans, we performed Multi-marker Analysis of GenoMic Annotation (MAGMA) competitive gene set analysis on 378,484 unrelated white European adults from the UK Biobank Study who had measures of quantitative ultrasound derived heel estimated BMD (eBMD) (Bycroft et al., 2018; Kemp et al., 2017; Morris et al., 2019). Gene-based tests of association detected significant gene-wide associations between eBMD and 71 of 520 osteomorph human orthologs (P_MULTI_ < 2.6 × 10^−6^; Figure 7K; Table S3). Notably, two of these genes, *Cadm1* and *Plxnb2* (plexin-B2), were deleted in mice in the OBCD database and shown to have skeletal phenotypes (Table S2). Furthermore, mutations in six of genes also caused monogenic skeletal disorders (*IARS2*, *DNMT3A*, *MMP14*, *ARSB*, *TBXAS1*, and *CSF1R*) in humans (Table S3). Despite these associations, competitive gene set analysis suggested that the set of osteomorph genes, as a whole, was not enriched for eBMD-associated genes. In summary, our analysis shows that osteomorph genes cause monogenic skeletal diseases and that some of these genes are also strongly associated with eBMD, indicating that osteomorph genes play a role in the pathogenesis of both rare monogenic and common polygenic skeletal diseases like osteoporosis.

## Discussion

Cell-to-cell fusion is a fundamental biological process that is involved in reproduction, development, and repair in multicellular organisms. Certain cells, such as skeletal muscle cells, exist in a fused multinucleated state to efficiently and synchronously carry out their function. Other cells, such as monocyte/macrophage lineage cells, are able to carry out their phagocytic function in a mononuclear state and only fuse under specific conditions when this is inadequate. For example, in granulomas, macrophages form multinucleated giant cells that attempt to “wall off” and contain intracellular pathogens, such as mycobacteria, and chronic irritants, such as foreign bodies, that cannot be eradicated or degraded (Anderson, 2000). In bone, monocyte/macrophage lineage cells fuse to form osteoclasts that resorb mineralized bone. It has been shown that multinucleation is required for efficient bone resorption (Pereira et al., 2018), and number of nuclei has been linked to bone resorption pit surface occupied by individual osteoclasts (Boissy et al., 2002). This is consistent with the intravital imaging of *in vivo* osteoclast dynamics in this study, which showed that osteoclasts resorb bone under steady-state conditions as shown by their uptake of fluorescent bisphosphonates. Stimulation with sRANKL promoted the fusion of these osteoclasts to form even larger polykaryons, potentially to increase their resorptive capacity.

The intravital imaging also revealed osteoclasts undergoing cell fission, in which large polykaryons separated into smaller daughter cells. This superficially resembles apoptosis, which has been reported in osteoclasts in response to external stressors, such as bisphosphonates (Hughes et al., 1995), estrogen (Hughes et al., 1996), and mechanical force (Kobayashi et al., 2000). Apoptosis has been demonstrated *in situ* in histological sections by TUNEL staining (Akiyama et al., 2005) and *in vivo* by laser scanning confocal microscopy in zebrafish (Chatani et al., 2011). These, together with *in vitro* studies of osteoclast cell fate, have led to the current dogma that the fate of an osteoclast is to die by apoptosis following completion of its bone resorbing activity (Boyce, 2013). However, our direct visualization of osteoclast dynamics *in vivo* did not reveal a single example of apoptosis in over 130 h of real-time intravital imaging. Only following deliberate induction of apoptosis by laser irradiation were apoptotic events captured *in vivo*. Notably, the morphological changes, subcellular size of the fragments, cellular dynamics, and tissue response during apoptosis were markedly different from those observed during cell fission. Accordingly, spontaneous osteoclast apoptosis is a rare event *in vivo* in contrast to the fission events, which were much more common.

Osteoclasts are able to migrate slowly over the surface of dentine slices and simultaneously resorb bone *in vitro* (Søe and Delaissé, 2017). However, the speed of resorbing osteoclasts *in vitro* is slow at <2 μm/min (Kanehisa and Heersche, 1988). Actively resorbing osteoclasts induced by sRANKL are polykaryons that may be too large to migrate coordinately and efficiently over the bone surface *in vivo*. The disassembly of osteoclasts by fission into smaller, more motile cells in one resorption pit may thus allow them to migrate more efficiently and then rapidly reassemble by fusion into functional osteoclasts at another site. Such a scenario would be consistent with the proposal that “primary” osteoclasts at the tip of the cutting cone can undergo fission followed by fusion with “secondary” osteoclasts in the reversal-resorption surface bone remodeling units (Lassen et al., 2017). In these scenarios, asymmetric distribution of cell adhesion molecules, chemokine receptors, and cytoskeletal proteins may contribute to cells budding off and moving in opposite directions, as we observed. Furthermore, separation of large polykaryons into smaller osteomorphs may allow them to persist and survive for extended periods of time until they are required again. This may explain the accumulation of osteomorphs in mice treated with OPG:Fc, and their rapid fusion to form actively resorbing osteoclasts upon OPG:Fc withdrawal. Studies examining interactions between osteoclasts, osteomorphs, osteoblasts, and osteocytes may reveal further insights into the cellular dynamics of bone turnover.

From a bioenergetic perspective, the recycling of osteoclasts during bone resorption conserves the energy cost of committing resources to recruiting precursors from the circulation and differentiating them into osteoclasts on the bone surface, resources which would otherwise be lost if osteoclasts underwent apoptosis. Moreover, when apoptosis was induced by two-photon photoablation, we observed an influx of macrophages to clear apoptotic debris, which is a further energy expenditure. Osteoclast recycling, and long-term retention of osteomorphs, represents an energy-efficient and rapid mechanism for osteoclast regeneration. Indeed, it was recently shown that osteoclasts were maintained by iterative fusion with circulating monocytic cells and that this resulted in longer-than-expected lifespan (Jacome-Galarza et al., 2019). This is consistent with our data and suggests that the fusion of osteomorphs with osteoclasts may also contribute to this increased lifespan.

Osteomorphs are formed by the fission of osteoclasts into smaller, more motile daughter cells that retain the ability to fuse to form functional osteoclasts. In contrast to osteoclasts that are attached to bone, osteomorphs are found in the bone marrow and blood. scRNA-seq analysis showed that osteomorphs, osteoclasts, and macrophage precursors were transcriptionally distinct cell states. The transcriptome of osteomorphs, compared to macrophages, was characterized not only by the upregulated expression of a number of canonical osteoclast genes (such as *Acp5*) but also genes not previously associated with osteoclasts (such as *Bpgm* and *Fbxo7*). Thus, osteomorphs are a cell type distinct from osteoclasts and their macrophage precursors.

Detailed phenotypic analysis of 40 mouse lines generated by the OBCD program showed that osteomorph upregulated genes have important roles in bone structure and function. Notably, 11 of the 17 gene knockout mice have not been previously annotated in the GO or MGI databases to have skeletal terms or bone phenotypes. Two of these (*Bpgm* and *Fbxo7*) were upregulated in osteomorphs but not osteoclasts, highlighting the potential of studying osteomorphs to reveal genes and pathways involved in bone homeostasis and disease. *BPGM* deficiency causes a rare human disease characterized by compensatory erythrocytosis due to a left shift in the haemoglobin-oxygen dissociation curve (Rosa et al., 1978). It will be interesting to determine if these patients also have a skeletal phenotype. Moreover, *Fbxo7* has been shown to positively regulate bone morphogenetic protein signaling (Kang and Chung, 2015), providing a potential mechanism for the skeletal phenotype in *Fbxo7* knockout mice. The clinical significance of osteomorphs to human disease is further exemplified by the finding that 23 osteomorph upregulated genes were involved in the pathogenesis of rare monogenic skeletal dysplasias and 71 were associated with eBMD. Together, these data indicate that osteomorphs and their genes may be implicated in the pathogenesis of rare monogenic skeletal dysplasias and common polygenic bone diseases such as osteoporosis.

A consequence of osteoclast recycling is that it allows a committed pool of cells to be maintained during periods of inactivity that are primed to respond rapidly to changing conditions. Indeed, inhibition of cell fusion by OPG:Fc disrupted recycling and resulted in the accumulation of osteomorphs, which were available to rapidly fuse and generate active osteoclasts following OPG:Fc withdrawal. While the scRNA-seq data showed osteomorphs expressed *Acp5* mRNA, we did not detect the accumulation of small osteomorphs with TRAP enzyme activity in the bone marrow by histomorphometry. This may suggest that while osteomorphs do not express TRAP protein at baseline, they are poised to express the protein when they are required to resorb bone again. This separation between *Acp5* gene and TRAP protein expression has also been described in osteocytes (Youlten et al., 2020). Regardless, osteoclast recycling provides a mechanism to explain the recent emergence of a rebound phenomenon, in which some patients have been reported to develop accelerated bone loss and rebound vertebral fractures 3–16 months after cessation of anti-RANKL treatment (Anastasilakis et al., 2017a; 2017b; McClung et al., 2017; Popp et al., 2016; Tsourdi et al., 2017; Zanchetta et al., 2018). It will be interesting to see if aberrant osteoclast recycling underlies skeletal diseases such as Paget’s disease and giant cell tumors, where there may be dysregulated cell fission/fusion cycles. Osteoclast recycling therefore not only provides a paradigm for understanding the behavior of these cells in their physiological niche *in vivo* but also a framework for understanding drug effects on bone homeostasis. Our study has revealed a suite of genes upregulated by osteomorphs, many of which have not been previously known to be involved in bone resorption. The bone phenotypes when these genes are deleted in mice and their role in monogenic skeletal dysplasias and association with eBMD confirm the important role of osteomorphs in bone homeostasis and disease and mark them as a valuable resource for future drug discovery.

## STAR★methods

### Key resources table

**Table:** 

REAGENT OR RESOURCE	SOURCE	IDENTIFIER
**Antibodies**		

Anti-mouse AXL-PeCy7	Thermo Fisher Scientific	Cat#: 25-1084-82; RRID:AB_2734852
Anti-Mouse CD16/CD32	BioLegend	Cat#: 101302; RRID:AB_312801
Anti-mouse CD193-BUV737	BD Biosciences	Cat#: 741699; RRID:AB_2871076
Anti-mouse CD74-BUV395	BD Biosciences	Cat#: 740274; RRID:AB_2740014
Anti-mouse CADM1	MBL International	Cat#: cm004-3; RRID:AB_592783
Anti-mouse CD11B-PerCPCy5.5	BioLegend	Cat#: 101228; RRID:AB_893232
Anti-chicken Dylight 680	Thermo Fisher Scientific	Cat#: SA5-10074; RRID:AB_2556654
Anti-mouse VCAM1-BV786	BD Biosciences	Cat#: 740865; RRID:AB_2740517

**Chemicals, peptides, and recombinant proteins**		

10x GeneAmp PCR buffer without MgCl	Thermo Fisher Scientific	Cat#: N8080010
AF647-Zol	BioVic	Cat#: BV501005 – 110 nmol
Alpha-MEM	GIBCO/Thermo Fisher Scientific	Cat#: 12571-603
Buprenorphine (Temgesic)	Sigma	Cat#: TEMG I
Calcium Chloride (CaCl_2_)	Sigma Aldrich	Cat#: C1016
Cathepsin K Fast 680	Perkin Elmer	Cat#: NEV11000
Clodronate	Sigma	Cat#: D4434
DAPI	Sigma Aldrich	Cat#: D9542
Dentine	Kind gift from Damian Myers, University of Melbourne	N/A
Dimethylformamide	Sigma	Cat#: D4551
EDTA	Sigma	Cat#: E5134
Foetal Calf Serum	Hyclone/Thermo Fisher Scientific	Cat#: 15-10.02 Cat#: SH30406.02
Glass bottom culture dish 35mm, 10mm, No.0	MatTek Corporation	Cat#: P35G-0-10-C
Glass bottom culture plate 10mm, 1.5 coverslip	MatTek Corporation	Cat#: P35G-1.5-10-C
Hoechst 33258	Sigma	Cat#: 33342
Ketamine	Mavlab Animal Health	Cat#: KETA M I
Ketaprofen	Troy Laboratories	Cat#: KETO II
Kollisolv® PEG E 400	Sigma Aldrich	Cat#: 6855
Marcaine	Astra Zeneca	Cat#: 2209748
Napthol ASBI posphate	Sigma	Cat#: D4551
Osteoprotegerin:Fc	Amgen Inc	N/A
OsteoSense 680EX Fluorescent imaging agent	Perkin Elmer	Cat#: NEV10020EX
Paraformaldehyde	Thermo Fisher Scientific	Cat#: ACR416785000
Pararosanaline hydrochloride	Sigma	Cat#: P-3750
PBS, pH 7.4	GIBCO/Thermo Fisher Scientific	Cat#: 10010049
Penicillin-Streptomycin (10,000 U/mL)	GIBCO/Thermo Fisher Scientific	Cat#: 15140148
Recombinant GST-RANKL 158-316 construct	Washington University School of Medicine, St Louis, USA	N/A
Recombinant GST-RANKL 158-316 construct	Oriental Yeast company	Cat#: 47197900
Recombinant GST-RANKL 158-316 construct	Amgen Inc	N/A
Recombinant Human CSF-1 (Human/Cynomologous MCSF/CSF-1 protein (His Tag) (100ug)	Sino Biological Inc	Cat#: 11792-H08H
RNasin® Plus RNase Inhibitor (40 U/ul)	Promega	Cat#: N2611
RPMI 1640 Medium, GlutaMAX™ Supplement	GIBCO/Thermo Fisher Scientific	Cat#: 61870036
Serum TRAP 5b ELISA – Mouse TRAPTM	Abacus ALS Pty Ltd	Cat#: IDSBTR103
Silicone Grease	Beckman Coulter	Cat#: 335148
Sodium Acetate buffer pH 2.5	Sigma	Cat#: S8750
Sodium Nitrite	BDH	Cat#: 30188
Soluble RANKL	Oriental Yeast company	Cat#: 471979000
Superase-In RNase Inhibitor (20 U/ul)	Thermo Fisher Scientific	Cat#: AM2696
Tartaric Acid	BDH	Cat#: T400-1009
T-putty	Thermagon Inc	Cat#: A10548-5
Wheat germ agglutinin, Alexa Fluor™ 488 conjugate	Invitrogen	Cat#: W11261
Xylazine	Troy Laboratories	Cat#: XYLA Z 2

**Critical commercial assays**		

ERCC ExFold RNA Spike-In Mixes	Ambion/Thermo Fisher Scientific	Cat#: 4456739
High Sensitivity D5000 ScreenTape & Reagents	Agilent	Cat#: 5067-5592 & 5067-5593
Nextera XT DNA Library Preparation Kit	Illumina	Cat#: FC-131-1096
Nextera XT Index Kit	Illumina	Cat#: FC-121-1012
Qubit dsDNA HS Assay Kit	Invitrogen/Thermo Fisher Scientific	Cat#: Q32854
SMART-Seq v4 Ultra Low Input RNA Kit for Sequencing	Takara Bio	Cat#: 634893

**Deposited data**		

ENSEMBL database	(Yates et al., 2020)	Via BiomaRt in R; Ensembl release 101, August 2020; Accessed November 2020
ISDS Nosology and Classification of Skeletal Disorders Database	(Mortier et al., 2019)	https://doi.org/10.1002/ajmg.a.61366
Mouse Genome Database	The Jackson Laboratory	http://www.informatics.jax.org, March 2020 release; Accessed November 2020
scRNA-seq data	This paper	SRA: SUB4871440
UK Biobank Study	(Bycroft et al., 2018)	https://doi.org/10.1038/s41586-018-0579-z

**Experimental models: organisms/strains**		

B6.129P2-*Lyz2*^*tm1(cre)lfo*^/J	Jackson Labs	JAX: 004781
B6.Cg*Gt(ROSA)26Sor*^*tm14(CAG-tdTomato)Hze*^/J	Jackson Labs	JAX: 007914
B6.129-*Rag1*^*tm1Mom*^/J	Jackson Labs	JAX: 002216
*Blimp-1*^Egfp/+^	Stephen Nutt, Walter and Eliza Hall Institute, Melbourne Australia	N/A
C57BL/6-Tg(Csf1r-EGFP-NGFR/FKP1A/TNFRSF6)2Bck/J	Jackson Labs	JAX: 005070
C57BL/6J	Australian BioResources	ABR: 000664
C57BL/KaLwRijHsd	Harlan	N/A

**Software and algorithms**		

BASiCS	(Vallejos et al., 2015; Vallejos et al., 2016)	https://bioconductor.org/packages/release/bioc/html/BASiCS.html
BiomaRt	(Durinck et al., 2009)	https://bioconductor.org/packages/release/bioc/html/biomaRt.html; RRID:SCR_019214
biovizBase	(Yin and Cook, 2020)	https://bioconductor.org/packages/release/bioc/html/biovizBase.html
CTAn	Skyscan	https://www.bruker.com/
Cutadapt	(Martin, 2011)	https://cutadapt.readthedocs.io/en/stable/; RRID:SCR_011841
Drishti-2.4	(Limaye, 2012)	https://github.com/nci/drishti; RRID:SCR_017999
FIJI	(Schindelin et al., 2012)	https://imagej.net/Fiji; RRID:SCR_002285
FlowJo	Tree Star	https://www.flowjo.com/; RRID:SCR_008520
Galene	(Warren et al., 2018)	https://doi.org/10.1371/journal.pone.0070687
ggbio	(Yin et al., 2012)	http://bioconductor.org/packages/release/bioc/html/ggbio.html; RRID:SCR_003313
ggfortify	(Tang et al., 2016)	https://github.com/sinhrks/ggfortify
ggplot2	(Wickham, 2016)	https://cran.r-project.org/web/packages/ggplot2/index.html; RRID:SCR_014601
Imaris	Bitplane	https://imaris.oxinst.com/packages; RRID:SCR_007370
NMF	(Gaujoux and Seoighe, 2010)	https://CRAN.R-project.org/package=NMF
MASS	(Venables and Ripley, 2013)	https://cran.r-project.org/web/packages/MASS/index.html
MAGMA	(de Leeuw et al., 2015)	https://doi.org/10.1371/journal.pcbi.1004219
MtrackJ	(Meijering et al., 2012)	https://doi.org/10.1016/b978-0-12-391857-4.00009-4
Osteomeasure	Osteometrics	https://www.osteometrics.com/
plink	(Chang et al., 2015)	https://www.cog-genomics.org/plink/
Prism	GraphPad	https://www.graphpad.com/scientific-software/prism/; RRID:SCR_002798
Scanco	SCANCO Medical	http://www.scanco.ch/; RRID:SCR_017119
R	R Project for Statistical Computing	www.r-project.org; RRID:SCR_001905
RSEM	(Li and Dewey, 2011)	https://github.com/deweylab/RSEM; RRID:SCR_013027
STAR	(Dobin et al., 2013)	https://github.com/alexdobin/STAR; RRID:SCR_015899
Vision DXA	Faxitron Bioptics/Hologic	https://www.faxitron.com/

**Other**		

Hypergeometric p value calculator	Graeber Lab	https://systems.crump.ucla.edu/hypergeometric

### Resource Availability

#### Lead contact

Further information and requests for resources and reagents should be directed to and will be fulfilled by the Lead Contact, Dr Tri Giang Phan (t.phan@garvan.org.au).

#### Materials availability

This study did not generate new unique reagents.

#### Data and code availability

The raw single cell data generated during this study are available at BioProject: PRJNA507938. Human genotype and phenotype data on which the gene set analysis was based, is available upon application from the UK Biobank (https://www.ukbiobank.ac.uk). Other data available upon reasonable request from the Lead Contact.

### Experimental Model and Subject Details

#### Ethical Statement

Animal experiments were performed in accordance with approved protocols from Garvan Institute/St Vincent’s Hospital Animal Ethics committee (ARA 14/43 and 18/33). Mouse studies at the Wellcome Trust Sanger Institute were performed under license by the UK Home Office in accordance with the 1986 Animals (Scientific Procedures) Act and the recommendations of the Weatherall report. Mouse experiments at the Wellcome Trust Sanger Institute were conducted under license (P77453634 and PPL80/2485) in accordance with the Animals (Scientific Procedures) Act 1986, recommendations of the Weatherall report and following approval of the Sanger Institute Animal Welfare Ethical Review Body.

#### Animals

##### Osteoclast and osteomorph experiments

LysM^Cre^ mice carrying a nuclear-localized Cre recombinase inserted into the *Lyz2* gene (004781; B6.129P2-*Lyz2*^*tm1(cre)lfo*^/J) (Clausen et al., 1999) were crossed with mice carrying the floxed tdTomato reporter (007914; B6.Cg-*Gt(ROSA)26Sor*^*tm14(CAG-tdTomato)Hze*^/J) (Madisen et al., 2010) to generate LysM^Cre^.tdTomato^LSL/LSL^ reporter mice in which cells of the myeloid lineage are red fluorescent. Blimp-1^gfp/+^ reporter mice carrying enhanced green fluorescent protein (GFP) inserted into the *Prdm1* gene (Kallies et al., 2004) were crossed with T and B cell-deficient *Rag1*^*−/−*^ mice (002216; B6.129-*Rag1*^*tm1Mom*^/J) (Mombaerts et al., 1992) to generate Blimp-1^gfp/+^.*Rag1*^*−/−*^ reporter mice in which cells expressing *Prdm1* are green fluorescent. In the MaFIA mice (005070; C57BL/6-Tg(Csf1r-EGFP NGFR/FKP1A/TNFRSF6)2Bck/J) (Burnett et al., 2004) cells expressing CSF1R are green fluorescent. C57BL/6J mice were either from the Australian Resources Centre (Canning Vale, WA) or Australian BioResources (Moss Vale, NSW). All mice were bred and maintained on a C57BL/6J background under specific-pathogen free (SPF) conditions. Animal experiments were performed at the Garvan Institute Biological Testing Facility. All animal holding areas in both facilities are maintained within a constant temperature of 21.4þC with humidity range to avoid animal stress and to minimize experimental variability. Lighting mimics 12 h day/night cycles to stimulate circadian rhythms. For mixed bone marrow chimeras, 7-20 weeks-old female mice were used as donors and 7-12 weeks-old female mice were used as recipients. Bone marrow reconstitution was confirmed by FACS analysis of peripheral blood and imaging and FACS-based experiments were performed 8-14 weeks later.

##### Interventional experiments *in vivo*

C57BL/KaLwRijHsd (BKAL) female mice (Harlan, Netherlands) were obtained from the Australian BioResources (Moss Vale, NSW). All mice were bred and maintained on a C57BL/KaLwRijHsd background under specific-pathogen free conditions. Animal experiments were performed at the Garvan Institute Biological Testing Facility. All animal holding areas in both facilities are maintained within a constant temperature of 21.4þC with humidity range to avoid animal stress and to minimize experiment variability. Lighting in animal rooms ensures 12 h’ light and 12 h’ darkness with a dawn / dusk simulation. 8-10 week old female mice were used for OPG:Fc and sRANKL treatment studies.

##### Skeletal phenotyping of knockout mice

The OBCD program performed high-throughput multiparameter skeletal phenotyping of mouse lines generated by the Wellcome Trust Sanger Institute Mouse Genetics Project (MGP) as part of the International Mouse Phenotyping Consortium (IMPC) (Bassett et al., 2012). Experiments were performed in accordance with ARRIVE guidelines. Mice were maintained in a pathogen-free unit under a 12 h’ light and 12 h’ dark cycle with *ad libitum* access to water and food (Mouse Breeders Diet, LabDiets 5021-3, IPS, Richmond, USA). Mice underwent a standardized primary phenotype screen (https://www.mousephenotype.org/impress) (de Angelis et al., 2015).

##### *In vitro* experiments

Primary culture experiments were carried out in a certified PC2 laboratory. Cell viability were determined using trypan blue and an automated cell counter counter (Thermo Fisher Scientific). All cells were maintained in a 5% CO_2_ incubator at 37°C and experiments were conducted in a Class 2 BioSafety certified cabinet.

### Method Details

#### Generation of mixed bone marrow chimeras

To generate osteoclast reporter mice, we lethally irradiated 7-12 weeks-old C57BL/6J recipient mice with 950-990 rads in split doses 6 h apart. Irradiated mice were rescued with 3-10 × 10^6^ bone marrow cells from *Lysm*^Cre/+^.*Tdtomato*^LSL/LSL^ and *Blimp-1*^Egfp/+^.*Rag1*^*−/−*^ or *Csf1r*^Egfp/+^ mice in a ratio of 1:4 for *Blimp-1*^Egfp/+^.*Rag1*^*−/−*^ and 1:1 for *Csf1r*^Egfp/+^ donors. Chimeric mice were analyzed 8-14 weeks later following bone marrow reconstitution.

#### sRANKL, OPG:Fc and clodronate treatment

Recombinant GST-RANKL158–316 (sRANKL) was produced using a bacterial construct provided by Dr. F. Patrick Ross (Washington University School of Medicine, St. Louis, MO). For *in vivo* experiments, sRANKL was administered at a dose of 1mg/kg i.p daily for 3 days or 2mg/kg i.p once. OPG:Fc (Amgen Inc) was administered at a dose of 10mg/kg i.p. twice weekly for 2 weeks or as a single treatment. Control mice received saline. Clodronate was administered at a dose of 30mg/kg i.p up to 3 times a week.

#### Intravital two-photon microscopy

Mice were injected via the tail vein with 50μl of either the quenched cathepsin K substrate (Cat K 680 FAST) 4 h before imaging, or the fluorescent inactive bisphosphonate OsteoSense 680EX (both from Perkin Elmer) 24 h before imaging. Intravital two-photon microscopy was performed on the tibia (Lawson et al., 2015). Mice were anaesthetized with 100mg/kg ketamine/5mg/kg xylazine and maintained on 1%–2% isoflurane supplemented with 100% oxygen. Mice were kept warm on a custom heated SmartStage (Biotherm) set to 38þC. An incision was made in the skin overlying the tibia and soft tissue resected. The exposed tibia was immobilised on a base of thermal conductive T-putty (Thermagon Inc.) and a waterproof seal made around the edges with silicone grease (Beckman Coulter). Imaging was performed on a Zeiss 7MP two-photon microscope (Carl Zeiss) powered by a Chameleon Vision II Ti-Sa laser (Coherent Scientific). Excitation wavelengths used were 870nm or 920nm to detect Cat K 680 FAST (Perkin Elmer) and OsteoSense 680EX and 920nm to detect tdTomato and GFP. Fluorescent images were acquired with a LBF 760 and BSMP 760 to enable detection of far-red signals. Non-descanned detectors were SP 485 (blue second harmonic), BP 500-550 (GFP), BP 565-610 (tdTomato) and BP 640-710 (Alexa Fluor 680). 425 × 425 × 150 μm imaging volumes were acquired with a W Plan-Apochromat 20 × /1.0 DIC (UV)Vis-IR water immersion objective at a resolution of 0.83 μm/pixel. Optical sections were acquired at 2-3 μm z-step intervals and time lapse images were acquired every 20-90 s intervals. Individual mice were imaged for up to 8 h. For induction of apoptosis, laser photoablation was performed with localized irradiation of a small region of interest (ROI) at 840nm with real-time registration of the photoconverted red signal (Chtanova et al., 2014).

#### Image analysis and cell tracking

Raw image files were imported into Imaris (Bitplane). All images were processed with a weighted Gaussian filter. In some cases, individual TIFF generated frames were then processed with a blind deconvolution program written in MATLAB following processing to avoid the ringing effect of deconvolution. The PSF of the image frame was retrieved during the deconvolution process. Files were reassembled in Imaris and further thresholds and filters applied where necessary. When required, sample motion in time series data were corrected using the open source package *Galene* (Warren et al., 2018). The three dimensional sample motion within each stack relative to a reference stack in the middle of the time series was estimated using an approach based on the Lucas-Kanade framework (Baker and Matthews, 2004), accounting for the microscope scan pattern. The displacement at 500 reference points evenly spaced through the data was iteratively refined using a trust-region algorithm, minimizing the difference between the current and reference frame. For alignment, only the stationary SHG or far-red channels were used. The data in each channel was resampled correcting for the motion to produce a time series aligned with the reference frame. Large volume datasets were broken up into smaller files and analyzed separately and subsequently reassembled. Cell fate mapping trees were generated by a blinded observer manually tracking each cell frame-by-frame, across multiple movies. Analysis of cell fate mapping trees allowed the number of fission and fusion events (/h) and the proportion of cells undergoing fission or fusion events (%) in each treatment group to be measured. Time-lapse images were exported, compiled and annotated in AfterEffects (Adobe), and movies converted using MacX Video Converter Pro v6.0.2, (Digiarty Software). For static data generation, each image analyzed was from an individual mouse. Surfaces generated based on the red signal in cells that co-express red, green and OsteoSense 680 in a semi-automated manner using the Create Surface function in Imaris, under the supervision of a blinded observer, to quantify their sphericity and volume. Data were exported into R and the MASS package (Venables and Ripley, 2013) was used to calculate the 2D kernel density estimation, data visualized using the ggplot2 package (Wickham, 2016) in R. The *FilamentTracer* module in Imaris was used to quantify observable cell processes and networks formed by cell-cell contracts with neighboring cells. While this tool may include migrating cells in a network with osteoclasts, direct examination showed these events were rare relative to the scale of the networks and did not affect the overall result. Contacts between LYSM^+^ mononuclear/macrophage cells and LYSM^+^CSF1R^+^ osteoclasts were manually tracked through individual frames by a blinded observer.

#### *In vitro* live cell imaging of osteoclasts

Primary mouse bone marrow macrophages were cultured directly on glass-bottom culture dishes (35mm Petri dish, 10mm microwell with no. 0 cover glass) (MatTek Corporation, Ashland, MA, USA) under osteoclastogenic conditions (100ng/mL RANKL and 25ng/mL M-CSF) for a period of 4 to 7 days. Prior to imaging, osteoclasts were incubated with wheat germ agglutinin, Alexa Fluor® 488 conjugate (Invitrogen, Carlsbad, CA, USA) or OsteoSense 680EX Fluorescent Imaging Agent (PerkinElmer Inc., Waltham, MA, USA), and Hoechst 33258 (bis-benzimide) (Molecular Probes Eugene, OR, USA) according to manufacturers’ instructions. Osteoclasts were imaged using the NIKON A1Si confocal microscope equipped with 20x (dry) and 60x (oil) immersion lenses (Nikon, Melville, NY, USA) under controlled atmospheric conditions (37°C and 5% CO_2_/air) in a Tokai Hit Stage Top incubator (INUG2E-TIZ, Fujinomiya-shi, Shizuoka-ken, Japan). Frames were captured every 2 or 10 min for 2 h or overnight using NIS-C Elements software (Nikon). Cell surface areas and cumulative cell trajectories were analyzed using FIJI and the MtrackJ plug-in (Meijering et al., 2012).

#### DEXA analysis of bone mineral density

Dual-energy X-ray absorptiometry (DXA) (Faxitron Ultrafocus DXA, Hologic, USA) was performed weekly or fortnightly on anaesthetised mice under 3%–5% inhaled isoflurane. Analysis of the hind limb was performed using Vision DXA (Hologic, USA) using a manually drawn region of interest encompassing the left hind limb to quantify Bone mineral density (BMD).

#### Micro-CT analysis of bone structure

Bones were scanned with a μCT scanner (Model 1172, Skyscan) at 50kV, 200mA with a 0.5mm aluminum filter using a pixel size of 4.3 μm. Images were acquired every 0.4þ through 180þ and analyzed with NRecon and CTAn software (Skyscan). 3D models were generated using the Drishti-2 tool (Limaye, 2012).

#### TRAP staining of osteoclasts

To identify osteoclasts, femora from mice were fixed in 4% PFA and decalcified in 0.34M EDTA before being processed to paraffin blocks. 5μm thick sections were stained for TRAP (Lawson et al., 2015). Osteomeasure (Osteometrics) was used to quantify osteoclast and osteoblast parameters on endosteal surfaces using standard methods. Analysis of osteoclast parameters on trabecular bone was quantified as number of TRAP+ multinucleated cells per unit area as mature trabecular bone surfaces were difficult to define due to retention of primary spongiosa bone in mice treated with OPG:Fc.

#### Measurement of serum TRAP 5b

Serum collected by retro-orbital bleeds, under isoflurane anesthesia, throughout animal phases were stored at −70þ C and then assessed for TRAP levels using the MouseTRAP (TRAcP 5b) ELISA kit (Immunodiagnostic System Ltd) following manufacturers guidelines.

#### *In vitro* osteoclast cell culture

Bone marrow cells were harvested from the mouse long bones by flushing with MEM, then cultured in 6mm diameter culture wells containing dentine slices (a kind gift of Dr Damian Myers, the University of Melbourne, Australia) in MEM/FBS with 25 ng/mL of M-CSF (University of Queensland Protein Expression Facility, St Lucia, Australia) and 0, 20 and 50ng/mL of recombinant RANKL (Oriental Yeast Company, Tokyo, Japan) in quintuplicate replicate cultures. Cells were cultured for 6 days; medium and mediators were replaced on day 3. Cells were then fixed and stained for TRAP (Quinn et al., 1999). Osteoclasts (defined as TRAP+ multinucleated cells containing ^3^3 nuclei) were then counted by brightfield microscopy (Leica DM4000). Imaging guide lines were scored onto the dentine surface using a scalpel and the whole surface scanned using a Leica DM6000 PowerMosaic to produce a high resolution (x20) tiled image. To detect and image the resorption pits, cells were removed from the dentine surface using chloroform/methanol extraction (3:1) than rubbed face down on smooth paper to remove attached debris. The dentine was then dehydrated through industrial methylated spirits and allowed to air-dry on Whatman paper then mounted on double-sided carbon tape and coated with 15 nm of carbon using a benchtop carbon coater (Agar Scientific). Disc surfaces were imaged in high vacuum (< 1 pascal) using a Vega scanning electron microscope (Tescan UK, Cambridge) software v4.2.24.0, and 4-quadrant back-scattered electron detector with software v1.1 (Deben, UK). Dentine slices were imaged then using the ImageSnapper automated image acquisition plug-in at a working distance of 15 mm with a 20 kV electron beam at 138–2000x magnification, with 3x frame averaging at scan speed 5. Images of the stained cells and SEM images of the pits on the same surface were then merged and carefully aligned using the scored guide lines in Adobe Photoshop.

#### *Ex vivo* osteoclast recycling

LYSM^+^CSF1R^+^ double-labeled cells were FACS sorted from the bone marrow chimeric mice and incubated *in vitro* in glass bottom 16mm diameter wells at ∼4 × 10^4^ cells per well containing unlabelled primary osteoclasts and stimulated to form osteoclasts with 100ng/mL sRANKL and 25ng/mL M-CSF in αMEM medium containing 10% FBS (with Glutamax and HEPES). Microscopy was performed at x20 on a Leica DMI 6000 SP8 microscope. Sorted LYSM^+^CSF1R^+^ cells were also plated onto established explant calvaria from 3- to 4-day-old Balb/C mice in 24-well plate wells with αMEM media. To enhance recycling osteoclast growth on calvaria, primary osteoclasts from C57BL/6J were differentiated on the calvaria cultures for 3-4 days with RANKL and M-CSF as above. 18,000 or 40,000 recycling cells were plated in 80 μL neat FCS onto each calvarium and left to attach at 37þC for 4 h before adding 0.5ml αMEM with 200ng/mL RANKL and 25ng/mL M-CSF in αMEM media. Cells were incubated for 6 days with media change at day 3 before staining with Hoechst (1:20,000 in PBS) and OsteoSense 680 (1:10,000 in PBS) and imaged in 10mm culture plates in PBS. Fluorescent images were acquired with a LBF 760 and BSMP 760 to enable detection of far-red signals. Non-descanned detectors were SP 485 (blue second harmonic), BP 500-550 (GFP), BP 565-610 (tdTomato) and BP 640-710 (Alexa Fluor 680). 425 × 425 × 150 μm imaging volumes were acquired with a W Plan-Apochromat 20 × /1.0 DIC (UV)Vis-IR water immersion objective at a resolution of 0.83 μm/pixel. Optical sections were acquired at 2 μm z-step intervals.

#### FACS analysis

##### Isolation of bone marrow osteomorphs

Bone marrow chimeric mice were injected with 3 doses of AF647-Zol (BioVinc, Pasadena, CA, USA) over 7 days prior to analysis. Mice were sacrificed, long bones were harvested and soft tissue was removed. Cells in the bone marrow were flushed from the long bones with PBS. Cells adherent to the bone were isolated from the remaining marrow-depleted bone fraction through two rounds of enzymatic digestion with collagenase A (2mg/mL) and dispase (2mg/mL) for 15 min at 37°C. Red blood cell lysis was performed using ammonium chloride prior to isolation of individual cells for flow cytometric analysis and FACS sorting.

##### Osteomorphs surface marker validation

Expression of osteomorph markers were assessed on viable bone marrow cells by multicolor flow cytometry (FACSymphony; BD Biosciences, North Ryde, Australia). Cells collected from the bone and marrow fractions were incubated in (1:200) Fc-block for 10mins and subsequently stained for 30mins on ice in the dark with the indicated antibodies. Cells were washed in FACS buffer (2% FCS in PBS) and stained with DAPI before analysis.

#### scRNA-seq library preparations

##### cDNA generation

Individual single-positive (LYSM^+^CSF1R^neg^ZOL^neg^ or LYSM^neg^CSF1R^+^ZOL^neg^) and triple-positive cells (LYSM^+^CSF1R^+^ZOL^+^) were FACS sorted from the marrow and bone surface of mixed irradiation bone marrow chimeras as above into 384-well plates containing 3μl of deposition buffer (0.9% Superase-In RNase Inhibitor (20 U/μl); 0.9% RNasin® Plus RNase Inhibitor 40 U/μl; Nuclease-free water; 0.05% 10x GeneAmp PCR buffer without MgCl). cDNA was generated and amplified for 22 cycles using the SMART-Seq v4 Ultra Low Input RNA Kit for Sequencing (Takara Bio) and steps were performed according to manufacturer’s protocol at half reaction volumes (Khoo et al., 2019).

##### Library preparation and sequencing

Libraries were generated using NexteraXT library prep kit (Illumina) using 1ng of input. Subsequent steps were performed according to manufacturer’s instruction at half reaction volumes.

Library pools were paired-end sequenced at 2x150 base pair on an Illumina NextSeq500 platform.

#### scRNA-seq data preprocessing and alignment

Library pools were de-multiplexed with the standard Illumina pipeline. Reads were trimmed twice by cutadapt (Martin, 2011) to remove NexteraXT indexes and SMARTer 3¢ CDS primer IIA and 5¢ IIA oligonucleotide sequences. Trimmed reads were mapped to mouse genome (mm10) reference library with the addition of *Egfp*, *Tdtomato* and ERCC spike-in control references. Alignments were performed using STAR 2.4.1 (Dobin et al., 2013) and expression count quantitated using RSEM (Li and Dewey, 2011) using default parameters.

#### scRNA-seq data normalization

Noise reduction, highly variable genes determination and differential gene expression (DGE) analyses were performed using BASiCS package (Vallejos et al., 2015; Vallejos et al., 2016) in R.

#### Determination of osteomorph upregulated genes

To determine the osteomorph upregulated genes, DGE analysis was performed by comparing LYSM^+^CSF1R^+^ZOL^+^ populations from the marrow fraction to the combined single-positive (LYSM^+^CSF1R^neg^ and LYSM^neg^CSF1R^+^) populations from the bone and marrow fraction.

#### Clustering analysis

Highly variable genes identified within the datasets were clustered using non-negative matrix factorisation (NMF) algorithm (Moran et al., 2018) using NMF package in R (Gaujoux and Seoighe, 2010).

#### Skeletal phenotyping of knockout mice

##### OBCD phenotyping methods

Anonymized left lower limb and tail samples from 16-week-old female wild-type and knockout mice were stored in 70% ethanol at 4þC, and randomly assigned to batches for rapid-throughput analysis (n = 2-6 mice per line). Overall, 19 skeletal parameters were determined for each individual mouse studied. Depending on the precise genetic background of the mutant line, parameters were compared to reference data obtained from either 320 16-weeks-old wild-type C57BL/6NTac female mice or 99 16-weeks-old wild-type C57BL/6Dnk female mice (Table S2). Coefficients of variation for each skeletal parameter were: femur BMC (2.0%) and length (2.1%); vertebra BMC (2.1%) and length (2.3%); trabecular bone volume/tissue volume (18.5%), trabecular number (7.3%), trabecular thickness (7.9%) and trabecular spacing (8.3%); cortical bone thickness (4.3%), internal diameter (6.0%) and BMD (4.0%); femur yield load (13.2%), maximum load (10.0%), fracture load (29.0%), stiffness (13.7%) and energy dissipated before fracture (26.7%); and vertebra yield load (13.0%), maximum load (10.3%) and stiffness (13.3%).

##### Digital X-ray microradiography

Following removal of soft tissue digital X-ray images were recorded at a 10 μM resolution using a Faxitron MX20 operating at 26kV (Qados, Cross Technologies plc, Sandhurst, Berkshire, UK). All lower limb and caudal vertebrae 6 and 7 were imaged with 1mm diameter steel, aluminum and polyester standards. Bone lengths and relative bone mineral content (BMC) were determined as previously described (Bassett et al., 2012). Briefly, lengths were determined using ImageJ calibrated with an X-ray image of a digital micrometre. To determine relative BMC, 2368x2340 16-bit DICOM images were converted to 8-bit Tiff images in ImageJ. This process used the modal gray level of the polyester standard to define gray level zero and the modal gray level of the steel standard to define gray level 255 in the final Tiff image. Increasing gradations of mineralization density were represented in 16 equal intervals by applying a pseudocolor lookup table. For each sample the median gray level (0-255) of the femur and caudal vertebrae 6 and 7 was determined.

##### Micro-CT Analysis

Cortical and trabecular parameters were determined using a Scanco uCT50 (Scanco medical, Zurich, Switzerland). Samples were scanned at 70kV, 200 μA, with a 0.5mm aluminum filter, 1 s integration time, no averaging, and images captured every 0.36° though 180° rotation. Reconstructions, ROI selection and analyses were performed using Scanco software. Trabecular bone parameters (Trabecular bone volume BV/TV, trabecular number Tb.N, Trabecular thickness Tb.Th, and trabecular spacing Tb.Sp) were calculated from scans performed at a voxel resolution of 5 μm in a 1mm region of the trabecular compartment beginning 100 μm proximal to the distal femoral growth plate. Cortical bone parameters (Cortical thickness Ct.Th, Internal endosteal diameter, and BMD) were calculated from scans performed at voxel resolution of 10 μm in a 1.5mm long region of mid-shaft cortical bone centered 56% along the length of the femur distal to the femoral head.

##### Biomechanical Testing

Biomechanical testing was undertaken using an Instron 5543 load frame (Instron Limited, High Wycombe, UK). Femur strength and toughness (yield load, maximum load, fracture load, stiffness, % energy dissipated prior to fracture) were derived from destructive three-point bend testing using a 50-N load cell and custom mounts with rounded supports. Femurs were positioned horizontally with their anterior surface upward between two mounting points with a span of 8mm. Load was applied vertically to the mid-shaft with a constant rate of displacement of 0.03mm/second until fracture. The biomechanical properties of caudal vertebrae 6 and 7 (yield load, maximum load and stiffness) were derived from compression testing using a 500-N load cell and two custom anvils. Individual vertebrae were bonded in vertical alignment to the lower custom anvil using cyanoacrylate glue and load was applied vertically at a constant rate of displacement of 0.03 mm/s until approximately 1mm of displacement had occurred (Bassett et al., 2012).

#### Osteomorph genes and skeletal ontology

To establish known associations of osteomorph genes to bone, genes defining osteomorphs were queried in the Mouse Genome Database (MGD), Mouse Genome Informatics, The Jackson Laboratory, Bar Harbor, Maine. World Wide Web (URL: http://www.informatics.jax.org, March 2020 release; accessed November 2020) and Gene Onotologies (GO) retrieved from the ENSEMBL database (Yates et al., 2020) using BiomaRt in R (Ensembl release 101, August 2020; accessed November 2020). String matching was performed using a list of bone-related terms (“bone,” “skele,” “osteo,” “chondro,” “cartil,” “clast,” “tooth,” “odont,” “dentin,” “joint,” “limb,” “ossif,” “suture,” “digit”) and further refinement to remove “cytoskeleton” and “muscle” strings from the descriptions from the MGD and GO databases to identify known bone-related associations to the osteomorph upregulated genes.

#### Osteomorph genes and rare skeletal disorders

Fisher’s exact test of independence was used in conjunction with *ISDS Nosology and Classification of Skeletal Disorders* database (Mortier et al., 2019) to investigate whether human orthologs of osteomorph defining genes were enriched for genes that cause monogenic skeletal disorders. The following parameters were used: (A) Successes in sample - the number of differentially expressed mouse genes that defined osteomorphs and that map to unique human orthologs that cause one or more skeletal disorders (either total or within each individual nosology-defined skeletal disorder groups). (B) Sample size - the number of differentially expressed mouse genes that defined osteomorphs within the population of genes for which gene expression was observed in any cell type, and that map to unique human orthologs. (C) Successes in population - the number of mouse genes for which expression was observed in any cell type, and that map to unique human orthologs that cause one or more skeletal disorders (either total or within each individual nosology-defined skeletal disorder groups). (D) Population - the number of mouse genes that are expressed in any cell type and that map to unique human orthologs. Estimates of enrichment were obtained using the online Hypergeometric p value calculator (https://systems.crump.ucla.edu/hypergeometric).

#### Osteomorph genes and associations with eBMD

Unique mouse ENSEMBL – human ENSEMBL orthologs of all protein-coding genes were mapped using BiomaRt in R (Ensembl release 75 - February 2014). MAGMA competitive gene-set analysis (de Leeuw et al., 2015) was used to investigate whether human orthologs of osteomorph defining genes were on average more strongly associated with quantitative ultrasound derived heel eBMD, than all other protein coding genes in the human genome.

##### Datasets used for analysis

MAGMA analysis was performed on a sample of 378,484 unrelated white European subjects (54% female, plink1.9 / 2 (Chang et al., 2015) pairwise kinship co-efficient < 0.044) from the UK Biobank Study (UKB) (Bycroft et al., 2018) that had valid eBMD measures and high-quality genome-wide HRC and 1000G/UK10K imputed data from the January 2018 release [(i.e., ∼17M genetic variants with an information quality score > 0.9, minor allele frequency > 0.05%, minor allele count > 1, genotyping hard call rate > 0.95, and weak evidence of deviation from Hardy-Weinberg equilibrium (p > 1x10^−6^)]. Details of how eBMD was defined and criteria for including individuals in the study are detailed in the original publications (Kemp et al., 2017; Morris et al., 2019).

##### Gene-based tests of association

Gene-based tests of association were conducted in MAGMA (v1.08) (de Leeuw et al., 2016) using imputed individual level genotype data for the analyses involving eBMD. Analyses involving eBMD were further adjusted for age, sex, genotyping array, and ancestry informative principal components 1 – 20. Gene-based tests of association encompassed a multi-model approach in which the association results from different gene analysis models were combined to produce an aggregate p value corresponding to the strength of evidence of association between each protein coding gene (+2kb upstream/1kb downstream) and eBMD. The three association models included: a principal components regression model, a SNP-wise mean χ2 model [*i.e*. test statistic derived as the sum of −log(SNP p value) for all SNPs that intersect the gene region of interest], and SNP-wise top χ2 model [(test statistic derived as the sum of −log(SNP p value) for top SNP in the region of interest)]. The aggregate approach was chosen as it yields a more even distribution of statistical power and sensitivity over a wider range of different genetic architectures. Importantly, due to incomplete genotyping coverage (i.e., no genetic variants were present in some genes), and gene level associations could only be estimated for 520 / 549 mappable osteomorph orthologs and 11,500 / 12,278 mappable orthologs with background expression and 16,754 / 18,184 of all mappable mouse – human orthologs.

##### Gene set analysis

Competitive gene set analysis was used to determine whether human orthologs of the 520 osteomorph defining genes was on average more strongly associated with eBMD than the all other protein coding genes in the genome. The analysis was conducted using default settings, and accounted for several confounding factors including: gene size, gene density (i.e., representing the relative level of Linkage Disequilibrium between SNPs in the gene) and the inverse of the mean minor allele count in the gene (i.e., to correct for potential power loss in very low minor allele count SNPs), as well the log value of these three factors.

### Quantification and Statistical Analysis

Statistical analysis was performed using Prism software (GraphPad) or in R. All data are presented as box-and-whisker plots with the mean with the whiskers representing ± 1.5 times of inter-quartile range. We used Mann-Whitney test or one-way ANOVA with Tukey’s correction for multiple comparisons.

## Supplemental information

Table S1. Genes upregulated by LYSM^+^CSFIR^+^ZOL^+^ osteomorphs in the marrow and LYSM^+^CSFIR^+^ZOL^+^ osteoclasts on bone, related to Figure 7

Table S2. Skeletal phenotypes of mice from the OBCD program with deletions in genes that are upregulated in osteomorphs, related to Figure 7

Table S3. Genes that define osteomorphs and their enrichment in skeletal dysplasias and association with eBMD, related to Figure 7
