# Osteoclasts recycle via osteomorphs during RANKL-stimulated bone resorption

**DOI:** 10.1016/j.cell.2021.03.010

**Published:** 2021-04-01

**Authors:** Michelle M. McDonald, Weng Hua Khoo, Pei Ying Ng, Ya Xiao, Jad Zamerli, Peter Thatcher, Wunna Kyaw, Karrnan Pathmanandavel, Abigail K. Grootveld, Imogen Moran, Danyal Butt, Akira Nguyen, Alexander Corr, Sean Warren, Maté Biro, Natalie C. Butterfield, Siobhan E. Guilfoyle, Davide Komla-Ebri, Michael R.G. Dack, Hannah F. Dewhurst, John G. Logan, Yongxiao Li, Sindhu T. Mohanty, Niall Byrne, Rachael L. Terry, Marija K. Simic, Ryan Chai, Julian M.W. Quinn, Scott E. Youlten, Jessica A. Pettitt, David Abi-Hanna, Rohit Jain, Wolfgang Weninger, Mischa Lundberg, Shuting Sun, Frank H. Ebetino, Paul Timpson, Woei Ming Lee, Paul A. Baldock, Michael J. Rogers, Robert Brink, Graham R. Williams, J.H. Duncan Bassett, John P. Kemp, Nathan J. Pavlos, Peter I. Croucher, Tri Giang Phan

(Cell *184*, 1330–1347.e1–e13; March 4, 2021)

As a result of an author oversight in the originally published version of this article, the name of an author, Alexander Corr, did not appear in the author list. This error has now been corrected in the article online, and the author contributions have been updated accordingly to reflect Alexander Corr’s contributions to the scRNA-seq analysis.

